# Unveiling the role of inorganic nanoparticles in Earth’s biochemical evolution through electron transfer dynamics

**DOI:** 10.1016/j.isci.2024.109555

**Published:** 2024-03-25

**Authors:** Xiao-Lan Huang

**Affiliations:** 1 Center for Clean Water Technology, School of Marine and Atmospheric Sciences, Stony Brook University, Stony Brook, NY 11794-6044, USA

**Keywords:** chemistry, inorganic chemistry, biochemistry

## Abstract

This article explores the intricate interplay between inorganic nanoparticles and Earth’s biochemical history, with a focus on their electron transfer properties. It reveals how iron oxide and sulfide nanoparticles, as examples of inorganic nanoparticles, exhibit oxidoreductase activity similar to proteins. Termed “life fossil oxidoreductases," these inorganic enzymes influence redox reactions, detoxification processes, and nutrient cycling in early Earth environments. By emphasizing the structural configuration of nanoparticles and their electron conformation, including oxygen defects and metal vacancies, especially electron hopping, the article provides a foundation for understanding inorganic enzyme mechanisms. This approach, rooted in physics, underscores that life’s origin and evolution are governed by electron transfer principles within the framework of chemical equilibrium. Today, these nanoparticles serve as vital biocatalysts in natural ecosystems, participating in critical reactions for ecosystem health. The research highlights their enduring impact on Earth’s history, shaping ecosystems and interacting with protein metal centers through shared electron transfer dynamics, offering insights into early life processes and adaptations.

## Graphical abstract

**Figure:**
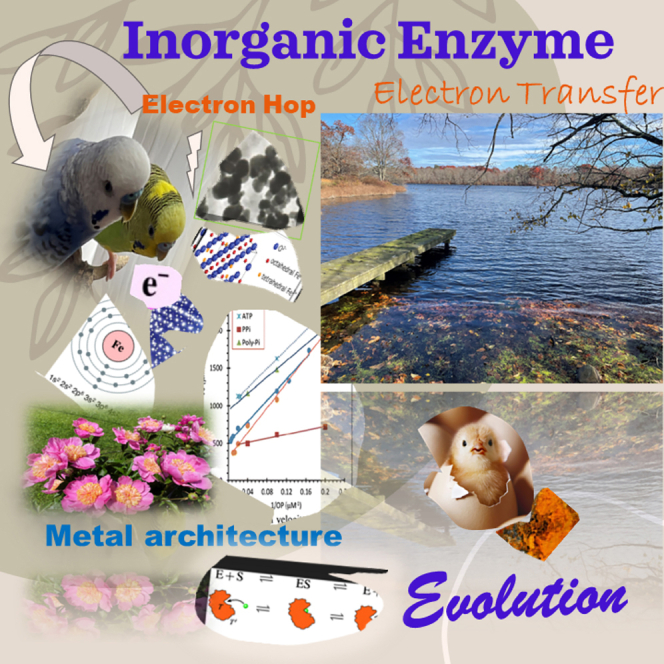

## Introduction

The oxidoreductases, key players in orchestrating electron transfer (ET) reactions across various organisms, are vital components in enzymatic activities.^1^^,^^2^^,^^3^ Their versatility is demonstrated through interactions with a wide array of substrates, both organic and inorganic, though they may generate reactive byproducts. Some oxidoreductases directly transfer electrons or utilize mediators such as cytochrome *c* to solid surfaces.^4^^,^^5^^,^^6^^,^^7^ This phenomenon, termed direct electron transfer (DET),^4^^,^^5^^,^^6^^,^^7^^,^^8^^,^^9^^,^^10^ was initially documented in 1977 by Eddows and Hill^11^ and Yeh and Kuwna.^12^ Their studies highlighted Cyc c’s reversible electrochemistry on gold and tin-doped indium oxide electrodes, observed through cyclic voltammetry. Notably, horseradish peroxidase (HRP)^13^ and laccase (Lc)^14^ also adhere to carbon electrodes, showcasing DET capacity. Impressively, more than 100 enzymes, with a predominant presence of oxidoreductases, are known to function under DET conditions.^7^^,^^15^^,^^16^^,^^17^^,^^18^^,^^19^^,^^20^^,^^21^ They play pivotal roles in metabolism, photosynthesis, and detoxification reactions. In the primordial conditions of the early Earth, oxidoreductases could have been crucial in primordial energy metabolism and the synthesis of biomolecules.

In 2007, Fe_3_O_4_ nanoparticles were found to exhibit peroxidase (POD)-like activity, giving rise to the field of nanozymes.^22^ Since then, nanozymes have gained significant attention in the fields of chemistry and biomedicine,^23^^,^^24^^,^^25^^,^^26^^,^^27^^,^^28^^,^^29^ finding applications in diverse areas, including medical, agriculture, and environmental protection. At the same time with another independent research, it was also observed that inorganic iron oxide nanoparticles can serve as inorganic phosphatase^30^^,^^31^^,^^32^ (Figure S1). This observation led to the proposal of the “inorganic enzyme hypothesis," suggesting that the metal architecture of inorganic nanoparticles bears similarities to proteins based on the experimental evidence.^31^^,^^32^^,^^33^

While the mechanisms of inorganic nanozyme continue to be debated,^34^^,^^35^^,^^36^^,^^37^ the notion that inorganic nanozymes should be considered inorganic enzymes has been gaining traction.^38^^,^^39^ This perspective underscores their role as biocatalysts and their potential contribution to the origin and development of life.

This review delves into inorganic nanomaterials as biocatalysts, focusing on their role as examples of inorganic oxidoreductases. It explores their involvement in ET processes, considering the relationship between the metal structure of nanoparticles and electron hopping, as well as the influence of the electron configuration of the metals involved. This sheds light on their potential as key players in biocatalysts and their relevance to the origin of life and its earliest evolution.

## Inorganic nanoparticles with intrinsic oxidoreductase activity

In 2007, Gao et al. made a groundbreaking discovery regarding the POD activity of Fe_3_O_4_ nanoparticles.^22^ These nanoparticles exhibited catalytic activity in the oxidation of various substrates, showing kinetics following Michaelis–Menten curves. The apparent Michaelis-Menten constants (K_m_) of HRP and 30 nm Fe_3_O_4_ particles measured with H_2_O_2_ as substrate are 3.70 and 154 mM, respectively. In contrast, the respective K_m_ values of HRP and 30 nm Fe_3_O_4_ measured with 3,3,5,5-tetramethylbenzidine (TMB) as substrate are 0.434 and 0.098 mM. The catalytic rates (k_cat_) of HRP and 30 nm Fe_3_O_4_ particles measured with H_2_O_2_ or TMB are 3.48 × 10^3^ and 8.58 × 10^4^ s^−1^ or 4.0 × 10^3^ and 3.02 × 10^4^ s^−1^, respectively.^22^ Thus, in terms of their catalytic efficiency (k_cat_/K_m_) the nanoparticles (H_2_O_2_: 560 mM^−1^ s^−1^; TMB: 3.1 × 10^5^ mM^−1^ s^−1^) are at least as good as their biological counterpart (H_2_O_2_: 940 mM^−1^ s^−1^; TMB: 9.2 × 10^3^ mM^−1^ s^−1^). Notably, the activity was related to the particle size. For nanoparticles with a diameter of 300 nm, the catalytic efficiency of a single nanoparticle was comparable to that of a single HRP molecule.^22^ Other inorganic iron oxide nanoparticles also displayed intrinsic peroxidase-like activity, although at a lower specific activity compared to HRP.^40^^,^^41^^,^^42^^,^^43^^,^^44^^,^^45^

Additionally, some iron oxide nanoparticles have exhibited multiple activities, including catalases (CAT), superoxide dismutase (SOD), and oxidases (OXD) activities associated with H_2_O_2_ and free radicals.^43^^,^^45^^,^^46^^,^^47^^,^^48^^,^^49^^,^^50^^,^^51^ Ferrihydrites, in particular, have been found to possess high intrinsic CAT-like activity and low intrinsic POD-like activity due to the abundance of hydroxyl groups in their nanocrystals^49^ (Figure S2). Actually, Ferrihydrites, functioning as inorganic CAT, have demonstrated *in vivo* their ability to produce O_2_ within the tumor microenvironment, leading to a substantial enhancement of the therapeutic effect of radiotherapy.^49^^,^^52^

Iron oxide nanoparticles were not the only inorganic nanomaterials to demonstrate oxidoreductase activity. Iron sulfide nanoparticles, including various phases such as FeS, Fe_1−x_S, and FeS_2_, were also found to catalyze peroxidase substrates, albeit with different kinetic parameters compared to HRP^53^^,^^54^^,^^55^^,^^56^ (Figure S3). For instance, Fe_7_S_8_ nanowires exhibited particularly high POD activity.^53^ The apparent K_m_ value for Fe_7_S_8_ NWs with TMB was determined to be 0.548 mM, significantly lower than that of Fe_3_O_4_ and even five times lower than that of HRP, indicating that Fe_7_S_8_ NWs have a higher affinity for TMB.^22^^,^^53^ Moreover, the K_m_ value for Fe_7_S_8_ NWs with H_2_O_2_ (0.895 mM) indicates a similar affinity for H_2_O_2_ compared to HRP and better H_2_O_2_ affinity than Fe_3_O_4_ nanoparticles.^22^^,^^53^ Furthermore, iron polysulfide nanoparticles (Fe_1−x_S and Fe_3_S_4_) displayed both POD and CAT activities, allowing them to break down H_2_O_2_ into free radicals and oxygen.^57^ These nanoparticles, especially Cys_0.5_-nFeS, demonstrated the highest activity.^57^

Density function theory (DFT) studies highlighted the importance of the architecture of inorganic nanomaterials in their catalytic activity (Figure S4). The mechanism for inorganic POD activity involves the chemisorption of H_2_O_2_ onto the nanomaterial surface, generating two hydroxyl adsorbates that undergo two reduction processes to remove the hydroxyl groups from the surface.^58^ The chemisorption energy and thus the POD-like activity are dependent on the metal architecture in the nanomaterials^58^ as will be discussed in more detail in the following section.

Importantly, it was found that even naturally occurring iron oxide nanoparticles in magnetosomes from magnetotactic bacteria (MTB) retained POD activity after removing the protein membrane,^59^^,^^60^ suggesting that the POD activity is an inherent property of the nanoparticles themselves. Biogenic iron oxide nanoparticles from *Burkholderia* sp YN01v (Fe_3_O_4_),^61^^,^^62^ *Comamonas testosterone* (Fe_1.44_O_0.32_(OH)_3.86_),^63^ and *Acinetobacter* strains (Fe_0.96_O_0.88_(OH)_1.12_)^64^ also exhibited intrinsic POD, SOD, and CAT activity (Figure S5A).

Ferritin, an iron storage protein present in various organisms, including archaea, bacteria, and eukaryotes, features an iron core that serves as a natural nano-structure with biogenetic iron minerals.^65^^,^^66^^,^^67^^,^^68^ Belarusian scientists found in 1999 that iron-containing crystallites in ferritins have POD activities,^69^ as biocatalysts, following Michaelis-Menten kinetics in the oxidation of TMB by hydrogen peroxide in acetate buffer solution (pH 4.2) in aqueous solution before the observation of Gao et al.^22^ Linear Lineweaver–Burk plots have been presented both for TMB (up to 3.0 mM) and H_2_O_2_ (up to 50 mM). The POD activity of the mineral core of ferritin was further confirmed with TMB, *o*-phenylenediamine (OPD), and N, N-diethyl-1,4-phenylenediamine (DPD) as substrates at 50°C in 2011^70^ (Figure S5B). Their experiments also indicated that the POD activity of horse spleen ferritin was derived from its ferric nanocore but not from the ferritin protein.^70^ A recent study on various ferritins from different biological groups potentially spanning different evolutionary lineages has further shown that these biogenic iron cores, after the removal of proteins from ferritins, exhibit enzyme-like activity (POD, CAT, OXD, and SOD) due to their metal structure, rather than the organic compounds that make up ferritins, particularly the amino acid sequences in these proteins (Figure S5C).^71^ For Archaea, the study examined ferritins from *Pyrococcus furiosus* (WP_011011871.1, labeled as pfFn), *Pyrococcus yayanosii* (WP_013905435.1, labeled as pyFn), and DNA protection protein from *Sulfolobus solfataricus* (NP_343470.1, labeled as ssDps). Bacteria were represented by Bacterioferritin (EcBfr) from *Escherichia coli* str. K-12 substr. MG1655 (NP_417795.1), Non-heme ferritin (EcFTn) from *Enterobacteriaceae* (WP_000917208.1), and DNA binding protein (EcDps) from starved *Escherichia coli* str. K-12 substr. MG1655 (NP_415333.1). Eukaryotic ferritins were represented by Heavy chain (HFn) from *Homo sapiens* (NP_002023.2) and Light chain (LFn) from *Homo sapiens* (NP_000137.2). This classification into different groups highlights the diversity of the iron cores from ferritins and their enzyme-like activity across various biological domains.^71^

Photoreactivation, the responsiveness of certain enzymes and inorganic nanozymes to light, plays a crucial role in various biological and chemical processes. This phenomenon is particularly prominent in oxidoreductases, enzymes involved in ET during redox reactions.^72^ PODs and OXDs containing iron-containing heme groups, such as HRP isozymes, demonstrate sensitivity to ultraviolet (UV) and blue light.^73^ The exact mechanisms behind this light-induced transformation remain uncertain, but aromatic amino acids such as tryptophan and tyrosine are implicated in electron tunneling processes.

This light-induced influence extends to inorganic nanozymes, including magnetite within MTB,^59^^,^^60^ α-Fe_2_O_3_ nanoparticles,^74^ TiO_2_ nanoparticles,^75^ Cu_2_Se hollow nanocubes,^76^ NH−MoO_3−x_ nanobelts,^77^ cobalt sulfide (Co_9_S_8_) nanodot,^78^ and CuO.^79^ For example, α-Fe_2_O_3_ nanoparticles exhibit enhanced substrate affinity and catalytic efficiency under light irradiation.^74^ Magnetosomes and naked magnetosomes also demonstrate heightened sensitivity to light, leading to improved substrate affinity.^60^ CuO nanorods show increased peroxidase-like activity under visible light, with a significant improvement in the generation of ⋅OH radicals^79^ (Figure S6). Composite nanoparticles such as TiO_2_ nanotubes coated with MoS_2_ nanoflowers (TiO_2_NTs@MoS_2_),^80^ plasmonic gold nanoparticle-modified Mn_3_O_4_ nanozyme (Mn_3_O_4_-Au),^81^ Fe_3_O_4_/Ag/Bi_2_MoO_6_ nanoparticle^82^ and quaternized chitosan (QCS)/silver (Ag)/cobalt phosphide (CoP) nanocomposites,^83^ all exhibiting photo responsive attributes. This characteristic allows for the modulation and enhancement of their catalytic activity using light energy.

Furthermore, the phenomenon of photoreactivation extends to near-infrared-II (NIR-II) light (1000–1700 nm). Cu_2_MoS_4_ (CMS) nanoparticles showcase both oxidase and peroxidase activities upon NIR-II light exposure.^84^ In the presence of NIR-II light, CMS NPs demonstrate OXD activity using L-ascorbic acid (AA) as a substrate. The K_m_ and V_max_ of CMS NPs for AA are determined as 12.06 μM and 0.11 μM s^−1^, respectively. Furthermore, CMS NPs display OXD activity with glutathione (GSH) and TMB as substrates.^84^ Under NIR-II light irradiation, GSH levels exhibit a decrease of more than 2-fold compared to reactions without light irradiation, indicating the enhanced OXD activity of CMS NPs. POD activity of Cu_2_MoS_4_ nanoparticles is also evident under NIR-II light. The K_m_ of CMS NPs for TMB and H_2_O_2_ is determined as 1.36 mM and 25.46 mM, respectively. The V_max_ of CMS NPs for TMB and H_2_O_2_ measures 27.29 × 10^−8^ M s^−1^ and 42.81 × 10^−8^ M s^−1^, respectively.^84^ Notably, the absorbance of the oxidized TMB product following treatment with CMS NPs and NIR-II light shows a 2-fold increase compared to CMS NPs alone, indicating the efficient acceleration of POD catalysis by NIR-II light.^84^ They demonstrate enhanced activity under NIR-II light, indicating the potential for utilizing this range of light in catalytic applications.

Cytochrome *c* (Cyt *c*) is a versatile protein found in a wide range of organisms, playing a crucial role in mediating ET during oxidative reactions.^85^ It belongs to the c-type Cytochrome family and is characterized by its distinctive CXXCH amino acid motif, which forms the binding site for the heme group embedded within the protein’s structure.^86^ Cyt *c* is primarily located in the inner membranes of mitochondria and chloroplasts, where it orchestrates electron transport chains essential for energy production.^20^^,^^87^ It shuttles electrons from oxidoreductases to terminal electron acceptor proteins or electrodes, facilitating processes such as ATP synthesis and chloroplast photosynthesis.^88^ The heme group (Haem) within Cyt *c*’s versatile active site enables its participation in various catalytic activities and ET reactions.^89^

In addition to its role in protein environments, Cyt *c* interacts with inorganic nanoparticles, exhibiting enzyme-like behaviors. For instance, when combined with WO_3_ nanoparticles, Cyt *c* demonstrates significant peroxidase-like activity, especially in the presence of water and hydrogen peroxide.^90^ Similarly, Cu_2_O nanoparticles mimic the functionality of the mitochondrial enzyme Cyt *c* oxidase,^91^ while CeVO_4_ nanoparticles serve as mitochondrial enzyme CcO, utilizing Cyt *c* as a biological electron donor in the four-electron reduction of molecular oxygen, thus integrating SOD-like functions.^92^ CdS nanorods accept electrons transferred from Cyt *c* as a bio-electron acceptor, catalyze the formation of superoxide anions, and exhibit NADH oxidase-like activity.^93^ Under physiological pH conditions, CdS nanorods, with the assistance of light, couple with dehydrogenase to recycle NADH.

Cyt *c* electron mediators are believed to have emerged around 3.5 billion years ago during the Archean era, a crucial epoch in Earth’s geological and environmental history. During this period, MTB demonstrated exceptional adaptations and biomineralization capabilities,^94^^,^^95^ utilizing specialized organelles known as magnetosomes to navigate using Earth’s magnetic field.^96^^,^^97^^,^^98^ These magnetosomes housed magnetic minerals such as magnetite (Fe_3_O_4_) and greigite (Fe_3_S_4_),^94^^,^^95^ with proteins containing the crucial CXXCH c-type Cytochrome-binding motifs integral to their structure.^95^^,^^96^^,^^99^^,^^100^^,^^101^ The Archean atmosphere contained significant concentrations of H_2_O_2_, presenting challenges for nascent life forms.^102^^,^^103^^,^^104^^,^^105^^,^^106^^,^^107^ Reactive oxygen species (ROS), including H_2_O_2_, could induce oxidative damage to cellular components. Defense mechanisms such as SOD, CAT, and POD evolved early to protect against ROS-induced damage across diverse domains of life.^108^^,^^109^^,^^110^^,^^111^^,^^112^

Laboratory investigations suggest that diverse iron oxide nanoparticles, precursors to magnetite, initially formed within MTB.^94^^,^^113^^,^^114^^,^^115^^,^^116^^,^^117^^,^^118^^,^^119^^,^^120^ These nanoparticles became encapsulated within lipid bilayer membranes, akin to structures found in mitochondria and chloroplasts.^121^ Iron oxide nanoparticles such as Fe_3_O_4_ and ferrihydrite could traverse lipid bilayers and enter cellular compartments without disrupting plasma membranes.^122^^,^^123^ The POD activity exhibited by magnetite within magnetosomes likely played a pivotal role in driving the formation of magnetosomes, as it conferred a survival advantage in environments rich in hydrogen peroxide.^59^^,^^60^^,^^108^^,^^109^^,^^110^^,^^111^^,^^112^ This “life fossil oxidoreductase” concept highlights the enduring role of magnetite nanoparticles within magnetosomes, acting as vital enzymes for ET in redox reactions.^38^ It suggests an adaptation to endure high hydrogen peroxide conditions and oxidative stress throughout Earth’s history.

Genetic analyses of magnetosome assembly reveal intricate processes that offer invaluable insights into the early evolutionary stages shaping life’s complexity.^124^^,^^125^ The convergence of Cyt *c* electron mediators and magnetosomes, coupled with the concept of the “life fossil oxidoreductase,”^38^ illuminates the innovative strategies adopted by early life during the Archean era to thrive amidst challenges such as hydrogen peroxide. This underscores the pioneering use of inorganic nanoparticles for survival and adaptation, ultimately contributing to the evolution and diversity of life forms we observe today.

In summary, inorganic nanoparticles, such as iron oxide, iron sulfide, and biogenic nanoparticles, exhibit significant oxidoreductase activities, including peroxidase, catalase, oxidase, and superoxide dismutase-like functions. Furthermore, certain nanoparticles demonstrate increased catalytic abilities in response to light, especially in the UV, blue, and NIR-II spectrum due to photoirradiation, along with the mediating effect of Cytochrome *c* for electron transfer. These groundbreaking concepts suggest that these nanoparticles serve as enduring biocatalysts crucial for redox reactions, particularly in early life and the emergence of life. These groundbreaking concepts suggest that these nanoparticles serve as enduring biocatalysts crucial for redox reactions, particularly in early life and the emergence of life. This adaptation allowed different life forms to thrive in environments with high levels of ROS, such as hydrogen peroxidase. This symbiotic relationship played a pivotal role in the various types of organisms' ability to thrive and adapt in environments characterized by heightened ROS levels. Together, these findings highlight the innovative utilization of inorganic nanoparticles for survival and adaptation on Earth, paving the way for the evolution and diversity of life forms we observe today.^39^

## Metal architectures in inorganic nanoparticles

The metal architecture in iron oxides is primarily determined by the ferric-ferrous composition (Fe (III)/Fe_total_) and the hydroxylation ratio (OH/Fe_total_)^126^^,^^127^^,^^128^ (Figure S7A). A fundamental structural unit of ferrihydrite and other iron oxide nanoparticles is the Back-Figges δ-Keggin cluster (Fe_13_), comprising 13 iron and 40 oxygen atoms^129^^,^^130^ (Figure S7B). Within this structure, central tetrahedrally coordinated Fe is linked to 12 peripheral octahedrally coordinated Fe atoms, forming edge-sharing groups of three through oxo bridges. Iron oxide nanoparticles between 2 and 6 nm can be visualized as a three-dimensional arrangement of these clusters, with adjacent clusters connected by various combinations of edge, corner, and face-shared octahedra, forming oxo bridges within the cluster^129^ (Figure S7C). The distance between Fe atoms depends on the type of connections, with corner sharing having the longest distance (3.39–3.70 Å) and face sharing the shortest (2.88 Å)^126^^,^^131^ (Figure S7D).

Magnetite possesses an inverse cubic spinel structure, where metal ions are distributed between tetrahedral and octahedral sites. Tetrahedral sites house Fe (III) ions, while octahedral sites contain both Fe (III) and Fe (II) ions in equal measure. This leads to the chemical formula Fe(III)_tetra_[Fe(II)Fe(III)]_octa_(O^2−^)_4_, indicating the position of ferrous and ferric ions within the structure^132^ (Figure S7E). Similarly, maghemite displays a spinel crystal structure with all iron cations in the trivalent state, and the charge neutrality of the cell is maintained through the presence of cation vacancies. The maghemite structure can be approximated as a cubic unit cell with the composition, ${\left({Fe}_{8}^{III}\right)}_{A}{\left[{Fe}_{\frac{40}{3}}^{III}{◻}_{\frac{8}{3}}\right]}_{B}O_{32}$,where ◻ represents a vacancy, A indicates tetrahedral, and B octahedral coordination sphere.^126^^,^^133^^,^^134^^,^^135^ In contrast, hematite possesses a primitive rhombohedral structure, with Fe (III) and oxygen atoms arranged differently, forming layers along the 3-fold axis in a hexagonal pattern.^126^^,^^136^^,^^137^^,^^138^In the crystal structure of trigonal hematite, the oxygen atoms are stacked in approximately closed-packed layers along the 3-fold axis. These layers are arranged in a hexagonal pattern.^139^

It is important to highlight that mixed-valent Fe minerals in the environment exhibit limited stability and undergo transformations through both abiotic and biotic pathways. Various factors such as oxygen levels, ROS, light, nitrate (NO_3_^−^), different forms of iron (II) and iron (III), and phosphorus concentrations can influence the transformation of iron oxide minerals^140^^,^^141^ or the metal architecture of iron oxide nanomaterials^126^^,^^128^ (Figure S8A). These external influences induce changes in the oxidation state, reactivity, and properties of iron, affecting its behavior in biological and biogeochemical processing.^126^^,^^142^^,^^143^^,^^144^^,^^145^ For instance, the study by Usman et al. demonstrates the transformation of ferric (oxyhydr)oxides, such as ferrihydrite, lepidocrocite, and goethite, into magnetite in the presence of Fe (II) ions.^146^ The reactivity and transformation sequence were found to be dependent on several factors, including the initial mineralogy of the oxyhydroxides, aging time, and solution chemistry. The order of reactivity for the transformation into magnetite was observed to be ferrihydrite > lepidocrocite > goethite^146^ (Figure S8B).

Solar irradiation also contributes to the formation of iron oxide nanoparticles. Even in the absence of oxygen in the atmosphere, low levels of ferric ions (Fe III) would have been generated at the ocean surface due to photooxidation.^147^ Solar irradiation promotes the transformation of ferrihydrite into goethite compared to dark conditions.^148^^,^^149^ For example, enhanced pathways of sunlight-driven ferrihydrite transformation in the presence of dissolved oxygen were observed, demonstrating the significant role of sunlight in the conversion of ferrihydrite to goethite.^149^ Photoinduced ET processes at the ferrihydrite interface generate redox active species, including hole-electron pairs, reactive radicals, and Fe (II). The production of hydroxyl radicals occurs through water oxidation, reduction of dissolved oxygen, and photolysis of Fe(III)-hydroxyl complexes^149^ (Figure S8C). Under acidic conditions, superoxide radicals act as oxidants for Fe (II) reoxidation, promoting the transformation of ferrihydrite. It is worth noting that the presence of inorganic ions such as chloride, sulfate, and nitrate not only influences the hydrolysis and precipitation of Fe (III) but also impacts the generation of radicals through photoinduced charge transfer reactions.^149^

Magnetite (Fe_3_O_4_) nanoparticles can be converted into maghemite (γ-Fe_2_O_3_) not only by oxidation with oxygen but also through various ions and/or ETs across the solid–solution interface.^150^ This involves the elimination of an electron from a surface ferrous ion, leading to the formation of a ferric ion and a cationic vacancy in the octahedral lattice, maintaining electrical neutrality. Electron mobility within the solid allows for the renewal of the ferrous sites at the surface, resulting in maghemite formation within the particles. At the same time, cationic vacancies are created by the migration of ferric ions to the surface, preserving the electroneutrality of the particles. The oxidation conditions significantly influence this process.^150^ Even in the absence of oxygen, 10 nm-sized stoichiometric magnetite particles (Fe (II)/Fe (III) = 0.5) in aqueous solutions over a biologically and environmentally relevant pH range (4–7) are still not stable. Fe (II) is released into the solution due to the H^+^-promoted dissolution process, leading to the partial oxidation of magnetite to a magnetite-maghemite solid solution.^151^ Under harsh conditions, such as exposure to a 0.07 mol HNO_3_ solution at 60°C in an air atmosphere, the process is much slower, with the complete oxidation of ferrous iron ions being observed, but the crystal phase of maghemite has not yet formed over 24 h.^152^ Moreover, recent research has revealed that when magnetite 111 is prepared under oxidizing or reducing conditions, the metal architecture at the surface, i.e., iron versus oxygen fractional surface terminations, is also different. A larger fraction of Fe-termination was found in the magnetite preparation under reducing conditions.^153^

When iron oxide nanoparticles interact with bacteria, especially with *Shewanella oneidensis MR-1*, a strain known for its metal-reducing abilities, remarkable transformations occur. This bacterium induces a significant reduction in hematite, resulting in the generation of substantial amounts of Fe (II). This process also leads to a noteworthy alteration in the crystalline structure of hematite, transitioning from a hexagonal to a cubic system. The bacterially catalyzed the reductive dissolution of hematite gives rise to intermediate states and the emergence of unique chemical environments characterized by Fe(II)/Fe(III) complexes with monodentate and bidentate coordination patterns. This transformative process involves the cleavage of iron-oxygen bonds within the hematite structure, resulting in the development of microstructures that form complexes with iron. These microstructures play a pivotal role in facilitating the creation of biogenic magnetite. Furthermore, observations from electron paramagnetic resonance (EPR) spectra indicate a diminishing EPR intensity over time, suggesting changes in the composition and local structures of both hematite and magnetite throughout the transformation process. DFT calculations further support the earlier observation regarding the octahedral configuration that arises as a consequence of Fe (II) production.^154^

In another study, Fe_3_O_4_ nanoparticles were exposed to a solution containing E. coli for 1 hour. As a result, approximately 46–48% of γ-Fe_2_O_3_ was generated, while only 22% of γ-Fe_2_O_3_ was generated in the control group (pure water, pH 5, 30°C). This was determined by comparing the changes in Fe−K-edge XANES spectra. No structural modifications of maghemite nanoparticles were observed, indicating that maghemite remained highly stable after direct contact with bacteria.^155^

Metal ion architecture in nanoparticles significantly influences inorganic oxidoreductase activities, particularly the POD activity of iron oxide nanoparticles.^49^^,^^156^^,^^157^^,^^158^^,^^159^^,^^160^^,^^161^^,^^162^^,^^163^^,^^164^^,^^165^^,^^166^^,^^167^^,^^168^ Fe_3_O_4_ nanoparticles demonstrate higher POD activity compared to γ-Fe_2_O_3_ and α-Fe_2_O_3_ nanoparticles.^169^ Notably, cycling catalysts can transform Fe_3_O_4_ to γ-Fe_2_O_3_, reducing ET rates and subsequently lowering POD activity. Specific POD activities for Fe_3_O_4_, α-Fe_2_O_3_, and γ-Fe_2_O_3_ nanoparticles are 1.79, 0.45, and 0.03 U·mg^−1^, respectively^169^ (Figure S9). The specific activity of Fe_3_O_4_ nanoparticles decreases over time, as supported by spectroscopic data.^169^ Prolonged reaction times lead to the gradual oxidation of interior Fe^2+^, affecting both surface and internal Fe^2+^ contributions to POD-like activity. Fe^2+^ ions within Fe_3_O_4_ nanoparticles facilitate ET to the surface, enabling sustained catalytic reactions. However, excess oxidized Fe^3+^ions migrating from the lattice act as the rate-limiting step, gradually diminishing the catalytic activity of the regenerated nanoparticles.^169^

The interplay between minerals and microbiology highlights the pivotal role of metal architecture in nanoparticles, a phenomenon widespread in nature. This is exemplified in the interaction between proliferating fungi and iron-rich hematite, resulting in the generation of biogenic ferrihydrite nanoparticles with inherent POD activity.^170^ Alterations in the non-lattice oxygen within the iron architecture largely contribute to their catalytic behavior, expanding our understanding of metal architecture’s influence on enzyme-like activity. Fungi actively mediate the catalytic reactions, confirmed by advanced microscopy techniques and biomass analyses^170^ (Figure S10). Another example involves incubating magnetite nanoparticles with *T. guizhouense*, resulting in a significant enhancement of their POD activity.^171^ Detailed spectroscopy analysis reveals changes in the metal architecture, particularly affecting non-lattice oxygen. This showcases nature’s ability to generate nanoparticles with oxidoreductase activity through modifications in their metal architecture. These examples illustrate the dynamic nature of metal architectures in iron oxide nanoparticles during fungal interactions. The interplay between fungi and iron oxide minerals leads to structural transformations directly impacting catalytic properties. This insight illuminates the intricate relationship between microorganisms and nanomaterials, emphasizing the role of metal architecture in dictating catalytic behaviors within these complex biological and chemical processes.

The study related to PVC dichlorination residues and iron chips treated with subcritical water revealed the presence of Fe_2_O_3_ nanoparticles in wastewater, demonstrating their intrinsic peroxidase-like properties driven by the unique metal architecture of iron oxide nanoparticles.^172^ This highlights the dynamic nature of metal architectures in iron oxide nanomaterials. Both ferrous and ferric ions within these nanoparticles occupy octahedral and tetrahedral coordinated interstitial sites within a closely packed anionic lattice of O^2−^/OH^−^. The distinctive properties of these nanoparticles arise from their electron configuration and oxygen coordination, including the presence of oxygen vacancies (OVs).^126^^,^^135^^,^^171^^,^^173^^,^^174^^,^^175^^,^^176^^,^^177^^,^^178^ This perspective extends to various iron sulfide nanoparticles. Much like iron oxide nanoparticles, diverse iron sulfide particles can undergo structural modifications due to chemical and microbial interactions.^179^^,^^180^^,^^181^^,^^182^^,^^183^^,^^184^^,^^185^^,^^186^^,^^187^^,^^188^ These interactions can lead to shifts in atomic and ionic arrangements within the nanoparticles, ultimately reshaping their metal architecture and influencing their properties. The metal architecture plays a crucial role in determining the functionalities and reactivity of these nanomaterials, making them remarkably adaptable, particularly in response to biological changes and catalytic behaviors, especially in the context of nanozymes. The interplay between iron oxide and iron sulfide nanomaterials with environmental factors and biological systems opens up new avenues for understanding the origins of life, the evolution of proteins, and their ecological functions. Examining these interactions sheds light on the fundamental processes that contributed to the emergence of life on our planet. Keep in mind that the story does not end with the elucidation of these static attributes. Nanoparticle structures are not rigid and unchanging entities; instead, they respond dynamically to a myriad of external influences, including both abiotic and biotic factors.

## Electron conduction mechanisms in inorganic nanoparticles

In solid-state physics, energy bands are crucial for understanding a material’s electrical properties. The valence band (VB) is the highest energy level filled with electrons involved in bonding.^189^ In contrast, the conduction band (CB) allows electrons to move freely, acting as charge carriers for electric current.^189^ Electron conductivity, or electrical conductivity, depends on how easily electrons move through a material.^190^^,^^191^ Electron mobility, which measures this ease of movement, and carrier concentration, the abundance of charge carriers, influence conductivity. Semiconductors and insulators are defined by their band gap, the energy difference between the valence and conduction bands.^189^ Materials characterized by elevated electron conductivity, featuring heightened electron mobility and carrier concentration, exhibit superior capabilities for effective ET, thereby catalyzing ET reactions adeptly. These materials hold paramount significance in shepherding the ET steps integral to biological functions and technological applications alike. Conversely, materials endowed with diminished electron conductivity may confront hindrances in expediting ET processes. In such scenarios, the pace or efficiency of ET reactions could wane, impairing the overall functionality of implicated systems.

In the realm of inorganic nanoparticles, the band gap plays a pivotal role in ET processes, determining the energy required for electron migration between the nanoparticle and other molecules or surfaces. ET is a fundamental process influencing interactions between inorganic nanomaterials and various organic entities, including large organic compounds such as proteins and DNA,^5^^,^^6^^,^^7^^,^^192^ as well as microorganisms.^4^^,^^193^^,^^194^ Numerous factors influence ET efficiency, with the band gap closely linked to the nanoparticles' electron configuration and structural characteristics, including size, structure, surface effects, composition, doping, and quantum confinement. This process holds significant sway over various biological mechanisms, especially in the realm of energy transfer within oxidoreductases from a physical standpoint.

To exemplify, the electron configuration of Fe (II) (iron ion with a +2 charge) and Fe (III) (iron ion with a +3 charge) is [Ar] 3d^6^ and [Ar] 3d^5^, respectively. In contrast, a neutral oxygen atom (O) assumes an electron configuration housing 2 unpaired electrons within the 2p subshell. These electron configurations contribute to a multifaceted electronic landscape within nanoparticles, fostering electron delocalization and mobility. Within the crystalline lattice, the transfer of electrons unfolds amid Fe (II) and Fe (III) ions, both inhabiting diverse oxidation states. The fusion of Fe 3d electrons with oxygen affords leeway for electron delocalization and mobility within the nanoparticle’s structural matrix. In turn, this underpins a phenomenon christened electron hopping—where localized electrons traverse between Fe (II) and Fe (III) ions. The impetus propelling this electron hopping derives from the oxidation state disparity between ions and the band gap, a metric representing the energy prerequisite for electron transitions.

The occurrence of electron hopping is postulated by theoretical models pertaining to myriad iron oxide nanomaterials, encompassing green rust,^195^ magnetite,^196^ iron oxyhydroxides,^197^^,^^198^ and hematite.^199^^,^^200^ Additionally, experimental observations have confirmed electron hopping on the surfaces of iron (oxyhydr)oxide,^201^ α-Fe_2_O_3_,^202^^,^^203^ and γ-Fe_2_O_3_^204^ NPs. The electron small-polaron hopping rate, quantifying the velocity of electron hopping, emerges as a pivotal factor impacting the kinetics of numerous iron redox reactions. This rate hinges on the short-range structural topology^201^ or lattice expansion of iron oxide,^202^ holding precedence over nanoparticle dimensions, suspension pH, or the infusion of multiple electrons per nanoparticle.^205^ These nuances underscore the central role played by the short-range structural topology and lattice expansion of iron oxide in orchestrating efficient electron hopping, highlighting the elemental role of electron hopping stemming from the nanoparticles' iron and oxygen electron configuration.

The electrical conductivity of iron oxide nanoparticles is subject to an array of influences, including the crystal structure and the spatial disposition of iron ions. Notably, charge-ordering (CO) emerges as a pivotal phenomenon underpinning electrical conductivity properties. Charge ordering denotes the organized arrangement of distinct metal oxidation states within a material’s crystal lattice. In the context of iron oxide nanoparticles, charge-ordering surfaces in magnetite at temperatures below the Verwey transition temperature of 120 K.^206^ Within iron oxide nanoparticles, the Fe-Fe distance within octahedral chains wields significant sway over charge ordering and electron mobility. The gap separating iron ions within these chains reverberates through the extent of charge transfer and electron hopping across different oxidation states, thereby wielding sway over the overall electrical conductivity of the material.

Both α-Fe_2_O_3_ and γ-Fe_2_O_3_ emerge as n-type semiconductors,^126^ denoting their capacity to conduct electricity through the orchestrated movement of electrons. Hematite’s band gap measures 2.2 eV, whereas maghemite’s slightly undercuts it at 2.03 eV.^126^ On the converse, wüstite (FeO) upholds status as a p-type semiconductor,^126^ with electricity conduction transpiring through hole movement, characterized by a band gap of 2.3 eV.^126^ In the scheme of things, goethite, lepidocrocite, and δ-FeOOH host comparably modest electrical conductivities in contrast to hematite and maghemite. Goethite exhibits a band gap of 2.10 eV, lepidocrocite clocks in at 2.06 eV, and δ-FeOOH boasts a band gap of 1.94 eV.^126^ An intriguing case manifests in magnetite, renowned for its multifarious electrical properties, embodying both p-type and n-type semiconductors within its structural confines. It features a minute band gap measuring 0.1 eV.^126^

Intriguingly, magnetite’s electrical conductivity, boasting a Fe (III)/total Fe ratio of 2/3, stands several multiples higher compared to hematite, characterized by a Fe (III)/total Fe ratio of 1.^207^ Magnetite operates as an electron conductor bearing a distinct spin orientation, while donning the mantle of insulator or semiconductor for electrons aligned with the opposite spin orientation. This unique property categorizes magnetite as a half-metallic material.^208^ Rigorous experimental scrutiny and density-functional calculations validate the presence of spin-split band energies, substantiating its half-metallic nature.^209^^,^^210^^,^^211^^,^^212^

Furthermore, investigations into sphere-shaped magnetite nanoparticles unveil shifts in electron mobility aligned with temperature shifts across an expansive range (173–373 K).^213^ These nanoparticles' electrical conductivity intertwines with AC frequency and temperature interactions, as evidenced in conductivity measurements (Figure S11A). The dielectric properties and AC conductivity at 273 K provide insights into the behavior of grain boundaries and grain conductivity, revealing distinct characteristics related to long-range and short-range mobility.^213^ These findings highlight the coexistence of two conduction mechanisms—barrier hopping and non-overlapping small polaron tunneling—across a range of temperatures and frequencies^213^ (Figure S11B). At low temperatures, the dominant mechanism governing the conductivity in Fe_3_O_4_ nanoparticles is charge carrier hopping, where electrons move between Fe (III) and Fe (II) ions. This mechanism involves two distinct modes of hopping, resulting in a smooth transition in conductivity as the temperature increases. At high temperatures, the conductivity mechanism still involves electron hopping between Fe(III) and Fe(II) ions, but the specific mode of hopping may undergo changes. These transitions between different modes of conductivity reflect the complex relationship between temperature and the structural dynamics of the nanoparticles, affecting the mobility of electrons within the nanoparticles. In essence, the transport of electrons is governed by the tunneling of small polarons, electron hopping, and movement between Fe (III) and Fe (II) ions.^213^

Additionally, the electrical conductivity of magnetite evolves in response to pressure alterations. Empirical studies divulge that under pressure ranging from 0 to 20 GPa, the resistivity of Fe_3_O_4_ plummets by over an order of magnitude, reaching a nadir. However, beyond 20 GPa, the resistivity resurges, ultimately doubling by 48 GPa^214^ (Figure S11C). This propensity for resistivity change underscores the pronounced susceptibility of Fe_3_O_4_’s electrical conductivity to shifts in pressure. Notably, the electronic attributes of Fe_2_O_3_ and FeO also undergo transformation under pressure and structural modifications.^215^^,^^216^^,^^217^

Temperature and pressure stand out as pivotal factors sculpting iron architecture in nanomaterials, accentuating alterations in Fe-Fe distance. These shifts, in turn, reverberate through the atomic structure of iron oxide nanoparticles, impacting the CO phenomenon and ET processes. The minimal Fe-Fe distance within the crystalline structure looms large as a critical factor dictating the nature and temperature at which charge-ordering transpires^218^^,^^219^^,^^220^^,^^221^^,^^222^^,^^223^^,^^224^ (Figure S11D). The electrical conductivity properties of iron oxide nanoparticles are further molded by the transmission of electrons between Fe (III) and Fe (II) ions. Adjustments to the Fe-Fe distance can impede or facilitate electron migration between these ions, invariably precipitating changes in electrical conductivity. Appreciating the nexus between the Fe-Fe distance, CO, and ET in iron oxide nanoparticles assumes pivotal significance in unraveling their electrical conductivity. Notably, shifts in the Fe-Fe distance—stemming from temperature and pressure shifts—unearth invaluable insights into the mechanics of electron transport, such as electron hopping and tunneling of small polarons. These shifts in the Fe-Fe distance stand as an inherent aspect of the metals' atomic structure and electron configuration, engendering repercussions for charge ordering and ET.

Moving on to iron sulfides, greigite (Fe_3_S_4_) exhibits characteristics akin to the mixed-valence ferrimagnetism observed in Fe_3_O_4_^225^^,^^226^^,^^227^ (Figure S12A). Pyrite (FeS_2_) acts as a semiconductor with a discernible band gap of 0.90 eV, displaying both p-type and n-type conductivity^228^^,^^229^ (Figure S12B). The electronic properties of pyrite are further influenced by the presence of sulfur vacancies, imparting notable effects on its conductivity.^230^^,^^231^ Pertinently, electron hopping materializes between Fe (II) and Fe (III) on octahedral crystal surfaces and between conductive sulfur-deficient grain cores ensconced within nominally stoichiometric FeS_2_^229^^,^^232^^,^^233^^,^^234^ (Figure S12C).

Fe_7_S_8_, distinguished by its monoclinic structure, showcases heightened magnetic anisotropy energy compared to FeS, making it a subject of keen interest in magnetism studies.^235^ It is worth noting that some iron sulfides, such as troilite, have demonstrated superconductivity below 4.5 K^236^^,^^237^^,^^238^ (Figure S12D). Furthermore, iron sulfides, much like their oxide counterparts, exhibit dynamic responses to changes in pressure^239^ and temperature.^240^ The crystal structure of iron sulfide (FeS) adopts a tetragonal configuration, with iron atoms coordinated by four equidistant sulfur atoms^241^^,^^242^ (Figure S12E). On the other hand, pyrrhotite (Fe_1-x_S) takes on a hexagonal crystal structure reminiscent of the NiAs arrangement.^235^ Other variations of Fe-S minerals, not mentioned, also contribute to the rich landscape of electronic behavior in this class of compounds. Additionally, it is important to consider iron deficiency in some iron sulfides, which can occur when the mineral lacks a sufficient amount of iron atoms.^235^^,^^239^^,^^240^ This deficiency affects the electronic properties of the material and can lead to alterations in its conductivity and magnetic behavior. Recognizing the impact of iron deficiency on the properties of iron sulfides introduces an additional layer of complexity to their behavior and electronic characteristics.

In summary, exploring ET mechanisms in inorganic nanomaterials, including both iron oxides and sulfides, reveals a complex tapestry of properties. These properties encompass band gaps, conductivity, and structural dynamics, all intricately linked to the electron configuration and coordination with oxygen or sulfur, including the various defects and vacancies. Importantly, these nanoparticle structures introduce an additional layer of complexity to the already intricate interplay of factors, being dynamic and responsive to external influences.

## Mechanisms of electron transfer in inorganic enzymes

In biological systems, ET is fundamental to reduction and oxidation reactions. Electrons play a central role, regardless of whether a biocatalyst or protein facilitates the process.^243^ Oxidoreductases, though not net electron producers themselves, excel at facilitating electron-transfer processes, making them vital in both biological and catalytic domains. Their essence lies in enabling electron flow.^244^^,^^245^

Oxidoreductases act as adept mediators, shuttling electrons either externally or within intricate intramolecular pathways—an event termed electron transport (ET_p_).^246^^,^^247^ This process primarily occurs through ionic channels within the biological milieu. The efficiency of electron transport within proteins correlates intimately with their electron-transfer proficiency and redox potential.^244^^,^^245^

To grasp the intricacies of the inorganic catalysis process, consider the essential role of electrons in biological processing. In many cases, the electron flux is low, and reaction rates are sluggish. In contrast to metal ions, which directly participate in these biochemical reactions, inorganic nanoparticles act as catalysts due to their unique structure and surface properties. Consider a scenario where a simple chemical equilibrium process involving electron transfer is established with a fixed equilibrium constant (K_eq_) for the biological reaction. While most of the electrons involved in the reaction are provided by the chemical constituents of the system, nanoparticles with electron hopping behavior can generate additional electrons, disrupting the chemical equilibrium governed by the fixed K_eq_. This disruption significantly enhances their role as catalysts, leading to increased electron flux and higher reaction rates. This phenomenon aligns with the second law of thermodynamics, which states that the disorder in a closed system always increases over time. For instance, Fe_3_O_4_ nanoparticles exhibit a distinctive POD activity by adsorbing H_2_O_2_ onto their surface, initiating redox reactions.^49^^,^^58^^,^^169^ This process differs from the conventional Fenton reaction, where the generation of hydroxyl radicals takes place in solution with metal ions.^248^^,^^249^ The pivotal step in hydroxyl radical generation with Fe_3_O_4_ nanoparticles involves redox reactions between H_2_O_2_ and the Fe (II) sites on the Fe_3_O_4_ surface.^250^^,^^251^ This orchestrated surge re-establishes equilibrium, injecting momentum into biological redox cascades, expediting metabolic processes. This symbolizes the essence of oxidoreductase functionality—a robust sustenance of ET flux, propelling the rhythmic cadence of reduction and oxidation. This interplay fosters a catalytic process fueled by the intrinsic electron hopping propensity of these nanoparticles distinct from conventional protein-based catalysis. as exemplified by HRP, wherein the genesis and retention of hydroxyl radicals transpire within the Fe-porphyrin ring throughout the ET sequence.^252^^,^^253^ The Fe (III) site nestled proximal to His170 and enveloped by a porphyrin architecture interfaces with Arg38 and His42, harmonizing to reorient H_2_O_2_.^253^^,^^254^

In addition to biological systems, a diverse array of inorganic nanomaterials, including transition metal oxides, sulfides, and selenides, exhibit remarkable electron transfer capabilities and hopping behavior. Some even demonstrate properties similar to those of superconductors, comparable to their inorganic counterparts such as iron oxides and sulfides, as we discussed earlier.

One fascinating category is that of inorganic enzymes, a special class of nanomaterials with protein-like catalytic activities.^38^ These biocatalysts, which may have predated all proteins or enzymes, perform functions akin to enzymes and display similar catalytic kinetics.^38^ For instance.

(1) Mixed-valence vanadium pentoxide V_2_O_5_ demonstrates semiconducting traits^255^ due to electron hopping dynamics within V^4+^ and V^5+^ ions,^256^ exhibiting intrinsic POD, OXD, CAT, glucose oxidase (GOX), and glutathione peroxidase (GP_x_) activity.^162^^,^^257^^,^^258^^,^^259^^,^^260^
(2) Mn_3_O_4_ nanoparticles demonstrate the polaron hopping of electron holes between Mn^4+^ and Mn^3+^ within octahedral sites,^261^ resulting in intrinsic CAT, GP_x_, and SOD activities.^164^^,^^262^
(3) MnO_2_ nanoparticles display direct electron hops within Mn - Mn chains,^263^^,^^264^ resulting in POD, CAT, OXD, and SOD activities.^265^^,^^266^
(4) Co_3_O_4_ NPs exhibit semiconducting attributes marked by Co^3+^-Co^2+^ hopping,^204^^,^^267^^,^^268^ concurrently demonstrating intrinsic POD and CAT activities.^159^^,^^269^^,^^270^^,^^271^
(5) Titanium dioxide (TiO_2_) nanoparticles, characterized by lower band gaps and electron hopping,^272^^,^^273^^,^^274^ also exhibit intrinsic POD activity,^275^ particularly with the photoelectron effect.^276^
(6) Nanocrystalline ceria (CeO_2_), with high electron conductivity and hopping attributes,^277^^,^^278^ can convert Ce^4+^ to Ce^3+^ due to oxygen vacancies,^279^ functioning as oxidoreductases (POD, CAT, OXD, SOD, nucleases, and photolyases),^161^^,^^165^^,^^280^^,^^281^^,^^282^ even phosphatase.^281^^,^^283^^,^^284^^,^^285^
(7) Cu_2_O/CuO nanoparticles, also known for their superconductivity,^286^^,^^287^ demonstrate intrinsic POD activity.^163^^,^^288^^,^^289^

In comparison to inorganic oxide nanoparticles, inorganic iron sulfide usually has a high electron transfer rate coupled with low resistivity, and even superconductivity as previously described,^236^^,^^237^^,^^238^ leading to improved corresponding enzyme activity.^53^^,^^54^^,^^55^^,^^56^^,^^57^ Additionally, molybdenum disulfide (MoS_2_) possesses both semiconductor properties^290^ and electron hopping behavior,^291^ allowing it to naturally act as POD, CAT, and SOD.^292^^,^^293^ Other nanoparticles such as α-FeSe, manganese selenide (MnSe), and molybdenum selenide (MoSe_2_) also demonstrate intrinsic POD activity^55^^,^^294^^,^^295^^,^^296^ due to their lower band gaps and electron hopping,^297^^,^^298^ including the superconductivity of α-FeSe.^236^^,^^299^^,^^300^

Moreover, certain inorganic nanoparticles, similar to proteinaceous enzyme, also respond to light, influencing their enzyme activity, including the light-sensitive α-Fe_2_O_3_ nanoparticles,^74^ TiO_2_ NP^75^^,^^276^ and various combinations thereof.^80^^,^^301^^,^^302^ The activity can also be influenced by nanoparticle modification, as seen with MoO_3_ nanoparticles. After modification, NH-MoO_3-x_ nanobelts exhibit sensitivity to light-induced electron dynamics, leading to changes in their band gap^77^ (Figure S13). Comparatively, MoO_3_ nanobelts, with a specific band gap of 2.592 eV, provide a reference point.^77^ NH-MoO_3-x_ nanobelts, with a reduced band gap of 1.793 eV, facilitate electron exchange through defect energy levels.^77^ This interaction not only improves electron conductivity but also triggers light-induced POD, OXD, and CAT activities, emphasizing the generation of ROS activity, creating a captivating interplay between temperature, energy bands, and catalytic responses. When these inorganic nanomaterials encounter light beyond their band gaps, an intriguing phenomenon unfolds. Electrons make a direct leap from valence to conduction levels, generating electron-hole pairs. Guided by photons and influenced by temperature, these pairs release surplus energy at the band’s edge, amplifying enzymatic efficiency. This orchestrated dance of electrons, orchestrated by both light and temperature, leads to an exhilarating enhancement of catalytic potency.

The introduction of Cyc-c electron mediators, akin to their protein counterparts, seamlessly woven into this intricate dance, stands as a testament to the multifaceted elegance inherent in these nanomaterials—an orchestration of catalytic prowess directed by the skillful hands of light, structure, and the enigmatic Cyc-c electron mediators. This remarkable contribution extends far beyond the domain of enzyme activity within synthesized nanoparticles. For instance, cadmium sulfide coupled with Cyc c serves as a bifunctional NADH oxidase and Cyc c reductase, showcasing the versatile roles that Cyc-c mediators play in orchestrating ET dynamics.^93^ Furthermore, CeVO_4_ nanozymes, mimicking the functions of the mitochondrial enzyme CcO, utilize Cyt *c* as a biological electron donor in the four-electron reduction of molecular oxygen, thus integrating SOD-like functions^92^ (Figure S14). These instances underscore the vast potential of Cyc-c mediators in influencing catalytic transformations.

**Figure 1:**
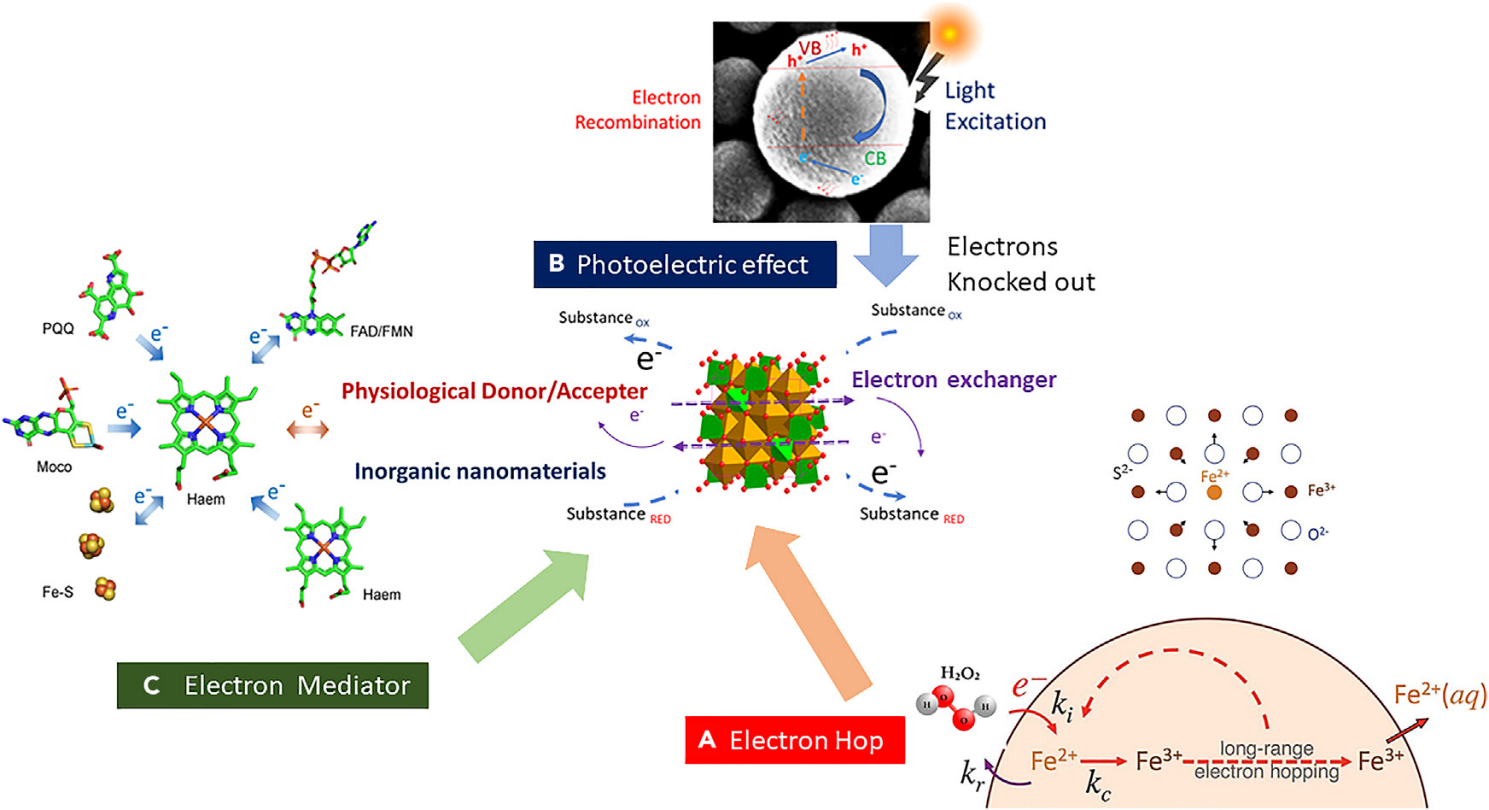
Electron Transfer Modes of the Inorganic Oxidoreductase (A) Electron Hopping in Inorganic Nanomaterials (Adopted from electron hopping behavior from iron oxide nanoparticles, reprinting from Katz J E et al.,^201^ copyright@2012, The American Association for the Advancement of Science), Multiples reports support this observation (green rust,^195^ magnetite,^196^ iron oxyhydroxides,^197^^,^^198^^,^^201^ hematite,^199^^,^^200^^,^^202^^,^^203^ γ-Fe_2_O_3_; ^204^ FeS_2_,^229^^,^^230^^,^^231^^,^^232^^,^^233^^,^^234^ Iron polysulfides (e.g., Fe_1-x_S),^235^^,^^239^^,^^240^ V_2_O_5_,^256^ Mn_3_O_4_,^261^ MnO_2_,^263^^,^^264^ Co_3_O_4_,^204^^,^^267^^,^^268^ TiO_2_,^272^^,^^273^^,^^274^ CeO_2_,^277^^,^^278^ α-FeSe,^291^ MnSe,^297^ and MoSe_2_^298^). (B) Photoelectron Effect and Photothermal Effect. Multiple reports support this observation.^59^^,^^60^^,^^74^^,^^75^^,^^76^^,^^77^^,^^78^^,^^79^^,^^80^^,^^81^^,^^82^^,^^83^^,^^84^^,^^276^^,^^301^^,^^302^ (C) Electron mediation by Cytochrome *c* (Adopted from Direct Electron Transfer of Enzymes Facilitated by Cytochromes and reprinting from Ma and Ludwing^87^) Multiple reports support this observation.^90^^,^^91^^,^^92^^,^^93^^,^^96^^,^^99^^,^^100^^,^^101^

This harmonious interplay extends its influence into uncharted horizons, particularly within the realm of the origin of life and its earliest evolution. In the opulent symphony of inorganic oxidoreductases, electron transfer modes come to the forefront, as illustrated in Figure 1. Here, electron hopping in the inorganic nanoparticles plays an essential role, with the interplay of light and Cyc-c offering a visual testament to the intricate pathways that pave the way for catalytic prowess. This visual representation vividly captures the essence of Cyc-c electron mediation, weaving threads of energy and transformation into the very fabric of these nanomaterials' reactivity.

Like the echoes of a symphony that linger long after the final note, the role of Cyc-c electron mediators reverberates beyond the laboratory, resonating through the corridors of scientific insight. It enriches our understanding of the delicate interplay between inorganic nanozymes and the grand theater of biochemical processes. In this grand symphony, electron hopping, coupled with the influence of light, finds its place as an essential and harmonious melody within the composition of nanoscale catalysis. Moreover, Cyc-c emerges as a bridge between the inorganic and organic worlds, particularly significant in the context of the origin of life, linking the elemental processes of early Earth to the intricate biochemical ballets of living organisms.

The influence of metal centers is deeply ingrained in biochemical transformations, dating back to the earliest stages of life, as exemplified by the compelling case of MTB.^94^^,^^95^ Remarkably, these orchestrations transcend the boundaries of inorganic nanoparticles, finding harmonious alignment with life’s ancient redox processes. This alignment is evident in the formation of biogenic iron oxide nanoparticles through biomineralization,^94^^,^^95^ referred to as “life fossil oxidoreductases,”^38^ where the POD activity^59^^,^^60^ and Cyc-c mediators play a pivotal role in modulating electron transfer pathways within the magnetite of MTB,^95^^,^^96^^,^^99^^,^^100^^,^^101^ as described in Section inorganic nanoparticles with intrinsic oxidoreductase activity.

All iron cores in ferritins can also be recognized as another example of “life fossil oxidoreductases."^38^ The observation of superoxide-diminishing activity in various biogenic iron cores of ferritin further emphasizes the significance of the metal structure in early life.^71^ After the removal of the protein, the iron core from prokaryotic ferritin, specifically both pfFn and pyFn from archaea *P. furiosus* or *P. yayanosii*, demonstrates higher SOD activity than that of eukaryotic ferritins, such as HFn and LFn from *H. sapiens*^71^ (Figure S5C). The iron/phosphate ratio in the iron core, a characteristic mainly determined by the structures of ferritins, also affects their SOD catalytic capability.^71^ The presence of phosphate in the iron core transforms it from a single crystalline structure to an amorphous iron phosphate-like structure, leading to a decreased affinity for the hydrogen proton of the ferrihydrite-like core. For example, ssDps, the DNA protection protein from the archaea *Sulfolobus solfataricus*, exhibits this behavior.^71^ Additionally, eukaryotes also have a higher phosphorus content due to evolutionary processes.^71^ These findings underscore the significance of the composition and evolution of ferritin structures in the context of early life and have potential implications for understanding the role of inorganic materials in biochemical processes.

The significance of these findings extends beyond synthetic experiments, reaching profound depths. In the synesthetic choreography of electron movement, these mediators establish a dynamic bridge connecting light, structure, and catalytic activity. This bridge not only unites nanomaterials and biochemical systems but also transcends temporal boundaries, linking the intricate dance of electrons in contemporary catalysis with the primordial rhythms of life’s redox processes.

The intricate dance of inorganic biocatalysts and their biological counterparts, proteinaceous oxidoreductases, forms a cornerstone of this narrative. These inorganic biocatalysts often exhibit striking parallels with their cognate oxidoreductases (proteinaceous enzymes)^13^^,^^14^^,^^15^^,^^16^^,^^17^^,^^18^^,^^21^ and universally possess DET capabilities.^7^ Even enzymes engaged in the hydrolysis of phosphate esters, known as phosphatases, share profound associations with protein-mediated ET processes.^303^^,^^304^^,^^305^ These phosphatases mirror the attributes of vanadium haloperoxidases, a subclass of oxidoreductases, embodying the essence of ET paradigms.^306^^,^^307^^,^^308^^,^^309^

The characteristics of biocatalysis (inorganic enzyme) are deeply rooted in the intrinsic metallic architecture properties of nanoparticles, including defects such as oxygen or metal vacancies,^310^^,^^311^^,^^312^^,^^313^^,^^314^^,^^315^^,^^316^ which are fundamental aspects of their nature. In particular, inorganic iron oxide and sulfur nanoparticles, with their complex metal architectures, exhibit unique electrical attributes. The electron configurations of Fe(III) and Fe(II) ions are distinct: Fe(III) is 1s^2^ 2s^2^ 2p⁶ 3s^2^ 3p⁶ 3d⁵, and Fe(II) is 1s^2^ 2s^2^ 2p⁶ 3s^2^ 3p⁶ 3d⁶. Within the atomic realm, the d orbitals of iron split into two sets with differing energy levels, a phenomenon known as d-orbital splitting, influenced by the crystal field effect from ligand interactions within coordination complexes. This energy difference governs the coloration and reactivity of iron compounds. The electron configuration of the oxide ion (O^2^⁻) is 1s^2^ 2s^2^ 2p⁶, with the additional two electrons in the 2p orbital. Similarly, the electron configuration of sulfide ions (S^2^⁻) is 1s^2^ 2s^2^ 2p⁶ 3s^2^ 3p⁶, with the additional two electrons also in the 2p orbital. This intricate interplay of electron arrangement unfolds through the coordination of iron’s electron configuration with oxygen or sulfur moieties, accentuated by vacancies, lending complexity to the ensemble. This interplay results in semiconductivity, characterized by small band gaps, facilitating efficient electron translocation. Additionally, it extends into superconductivity, akin to pyrrhotite. This intrinsic proclivity for spontaneous electron hopping manifests as a dance across octahedral surface locales, choreographed by Fe 3d electrons in partnership with oxygen or sulfur partners. In essence, the symphony of electron configurations, orbital interactions, and atomic vacancies composes a harmonious score, producing the electrical attributes of inorganic iron oxide and sulfur nanoparticles. This resonant interplay showcases the elegance of atomic structures and their emergent properties, shaping nanoscale reactivity and catalysis.

It is noted that electron hopping, where electrons move between localized sites within a material, is fundamental and observed in both inorganic nanoparticles and biomacromolecules such as proteins and DNA.^317^ In inorganic nanoparticles, this process occurs within the crystal lattice, influenced by factors such as particle size, shape, and surface chemistry. For example, smaller nanoparticles may exhibit enhanced electron hopping due to pronounced quantum effects. In biomacromolecules, electron hopping is vital in various biological processes.^247^^,^^318^ Proteins, with their complex structures, facilitate electron transfer through specific amino acid residues or cofactors. Metal ions or organic cofactors within proteins can act as electron carriers, allowing electrons to move along redox-active sites. Similarly, DNA, despite its role in genetic information storage, exhibits electron hopping behavior under certain conditions, influencing processes such as DNA repair and oxidative damage.

The mechanisms underlying electron hopping in both inorganic nanoparticles and biomacromolecules are intricately linked to their structures and characteristics. In inorganic nanoparticles, the arrangement of atoms and the presence of defects or impurities affect electron hopping. In biomacromolecules, the specific arrangement of amino acids or nucleotides, as well as the presence of metal ions or organic cofactors, modulate this process. Understanding these mechanisms is essential for elucidating the role of ET in biological systems and for designing functional materials with tailored electronic properties. Moreover, comprehending the connection between the metal center structure of proteins and the metal structure in inorganic nanoparticles is crucial for understanding the evolution and emergence of life.^39^ This dynamic relationship has profound implications for biological functions and technological applications, potentially shaping the course of evolution on Earth.

## Bridge of inorganic nanoparticles in biomolecular evolution

In our journey back to the conditions of early Earth, a spotlight is cast upon the establishment of fundamental biomolecules that have left indelible marks in the annals of our planet’s history. Among these molecules, primitive amino acids,^319^^,^^320^^,^^321^ adenine,^321^^,^^322^ nucleobases,^323^ nucleotides,^324^^,^^325^ sugars,^326^^,^^327^ lipids,^328^^,^^329^ and thiodepsipeptides^330^ stand as the cornerstones of this biochemical narrative. In this intricate interplay, amino acids emerge as protagonists, serving as the elemental building blocks of proteins and playing a pivotal role as conduits for ET processes, particularly within the realm of oxidoreductases. These amino acids boast diverse functional groups, ranging from amines to carboxylic acids, each eagerly participating in the choreography of ET reactions.

Amidst this intricate dance, the stage is set for the introduction of key electron relays—tryptophan and tyrosine residues. These entities come forth as maestros of electron flow, orchestrating efficient electron hopping and rapid electron tunneling across substantial distances within the intricate folds of metalloenzymes.^331^^,^^332^^,^^333^^,^^334^^,^^335^^,^^336^ As the dance continues, an additional layer of complexity is woven into the narrative—the interplay of light activation. This interplay introduces a symphonic interlude, where photons harmonize with matter, enriching the catalytic activity of enzymes and contributing to the captivating intricacies of biochemical transformations.

Within this ensemble, certain proteins stand out as virtuosos, housing heme groups with iron at their core. Consider POD,^253^^,^^337^^,^^338^ CAT,^339^^,^^340^ Fe-SOD,^341^ and cytochrome P450 peroxygenase,^342^^,^^343^ as prime examples. Together, they collaborate to construct the iconic Fe-oxo-Fe bridge, a structural marvel crucial to the catalytic procession of metal centers. This structure assumes a pivotal role in the delicate ballet of electron acceptance and donation during redox reactions.^344^^,^^345^^,^^346^ Through their intricate coordination, they imbue the dance of electrons with grace and purpose, ushering in moments of transformation that define the very essence of life’s processes.

The journey of functional groups extends beyond nature’s enzymatic boundaries, venturing into the realm of nanomaterials. While the selection of amino acids by humans for nanomaterial modification showcases deliberate innovation, the natural selection of amino acids within proteins through evolution is a result of intricate biological processes. This diverse array of amino acids and their arrangement, i.e., proteins, which has been shaped over millions of years, represents one of the nature’s greatest products—driving the complexity and functionality of living organisms. Precise nanomaterial modification with amino acids or other functional groups illustrates human ingenuity, yielding heightened ET rates and catalytic efficiency.^347^^,^^348^^,^^349^^,^^350^^,^^351^^,^^352^^,^^353^^,^^354^^,^^355^^,^^356^^,^^357^^,^^358^^,^^359^^,^^360^^,^^361^^,^^362^^,^^363^^,^^364^^,^^365^^,^^366^^,^^367^ These adept adjustments amplify surface interactions, culminating in more efficient ET processes. A notable example of this symphony is evident in histidine-modified Fe_3_O_4_ nanozymes, where a significant increase in apparent affinity K_m_ for the substrate H_2_O_2_ leads to a remarkable 20-fold enhancement in catalytic efficiency k_cat_/K_m_.^347^ Furthermore, research involving irony polysulfide nanoparticles (Fe_1−x_S and Fe_3_S_4_) unveils POD and CAT activities.^57^ Even when molybdenum trioxide nanoparticles reveal limited inherent electron conductivity,^368^ the introduction of triphenylphosphonium ions emerges as a masterful orchestrator, directing the activities of sulfite-oxidizing enzymes with precision and finesse.^369^ Similarly, the strategic utilization of near-Infrared-II (NIR-II) light unveils its transformative power, deftly lowering the bandgaps of NH−MoO_3−x_ nanoparticles. This nuanced reduction in bandgaps acts as a catalyst, triggering the liberation of electrons and catalyzing the remarkable functions of POD, CAT, and OXD.^77^

The formation and role of the metal centers stand as pivotal chapters intricately intertwined with the broader fabric of metalloenzymes structure and function. Nestled within the oxidoreductase protein, the metal center is not a passive participant but rather a testament to the symphonic interplay between its structural stabilization by amino acid sequences and its catalytic prowess within the Fe-oxo-Fe architecture.^38^^,^^39^ This catalytic potential, however, does not arise in isolation; it is accentuated by a harmonious ensemble of hydrogen bonds meticulously woven among the protein’s amino acids. This ensemble acts as a stabilizing force, amplifying the catalytic capabilities of the metal center to unprecedented heights. These insights resonate deeply, highlighting the crucial role of functional groups, particularly amino acids, in further fortifying the stability and selection of metal centers in metalloenzymes. Their impact reverberates across the secondary and tertiary levels of protein structure, where they engage in a seamless interplay with the evolving frameworks of metalloenzymes. Like skilled artisans, these functional groups assume the role of stabilizers—a concept elegantly championed by Huang.^38^^,^^39^ Yet, their contributions extend beyond mere stability; they lay the very foundation upon which the early pathways of life were etched, igniting the emergence of catalytic processes that have intricately woven the tapestry of life as we know it today.

The evolution of metalloenzymes has often been spotlighted through the lens of primary and secondary protein structures.^334^^,^^335^^,^^336^^,^^370^^,^^371^^,^^372^^,^^373^ This focus heavily relies upon gene codes or DNA, casting somewhat limited light on tertiary/quaternary structures and the integral metal centers. Within these tertiary structures, an enigma takes root—an enigma of metal center formation. While many studies have delved into the intricacies of folds, a significant emphasis has been placed on the secondary structure,^374^^,^^375^ often relegating metal centers and tertiary structures^376^^,^^377^^,^^378^ to the shadows. This fact suggests that the metal center could be considered a primary determinant in the early stages of protein folding, potentially preceding the involvement of amino acid residues in stabilizing the protein’s structure.^38^^,^^39^ This hypothesis aligns with the intricate relationship between inorganic nanoparticles, metal centers, and protein folding, highlighting the significance of metal ions or inorganic nanoparticles in shaping the functional properties and evolution of metalloproteins.

The story of ET and its evolution remains partially obscured, despite acknowledgments of the role played by iron-sulfur cluster proteins as electron carriers and transfer agents. Amidst these uncertainties, the pivotal question lingers—did metal centers precede primary and secondary protein structures, or did they arise in tandem or even later in the evolutionary journey? Does the formation of these metal centers intertwine with DNA/RNA synthesis, or does it emerge as a distinct process^38^? The selection of metals within metalloenzymes appears to be intricately woven with the fabric of geochemical processes, rather than solely being dictated by genetic materials such as DNA or RNA.^249^^,^^379^^,^^380^^,^^381^^,^^382^^,^^383^^,^^384^^,^^385^

A pivotal study conducted by Eck and Dayhoff in 1966 heralded a paradigm-shifting concept, unveiling the remarkable potential of ferredoxin to harness photon energy even in the absence of a fully established genetic code.^386^ This extraordinary attribute emanated from its inorganic active sites—clusters of [FeS]—and its elegantly simple, repetitive amino acid sequences. Subsequent experiments and computational models lent credence to this audacious hypothesis, suggesting the birth of a protoferredoxin, characterized by [2Fe-2S] and [4Fe-4S] motifs. These motifs emerged through the photooxidation of ferrous ions under UV light, facilitated by the duplication of an iron-sulfur tripeptide motif.^387^^,^^388^ The significance of ferredoxins spans the spectrum of life, from ancient anaerobic bacteria to sophisticated plants and animals, making them invaluable subjects for studying the evolution of metalloproteins.^389^^,^^390^^,^^391^

Weiss et al.'s pioneering research brilliantly illuminated the prevalence of FeS clusters and radical reaction mechanisms within the last universal common ancestor (LUCA), the progenitor of all cellular life forms.^392^ This revelation provided a tantalizing glimpse into the early utilization of FeS clusters as trailblazers that facilitated ET and orchestrated redox reactions within the nascent cells of the primordial world. Simultaneously, explorations into the enigmatic realm of hydrothermal vents, often considered the cradles of life’s origin, unveiled remarkably similar observations.^393^^,^^394^^,^^395^ Notably, the metal centers of Fe-S cluster proteins and diverse iron sulfides exhibited intriguing parallels, showcasing the elegant design of nature^396^^,^^397^^,^^398^ (Figure S15). For instance, the [2Fe 2S] motifs adorning eukaryotic ferredoxins and Rieske proteins displayed a symphony of coordination between paired iron atoms and two equidistant sulfur atoms.^399^^,^^400^ In contrast, the quartet of iron atoms, harmoniously coordinated with four equidistant sulfur atoms in high-potential iron-sulfur proteins and iron regulatory proteins (IRPs), composed the resonant tune of [4Fe 4S].^401^^,^^402^^,^^403^^,^^404^ Rubredoxin, with its graceful choreography, highlighted a single iron atom partnered with four equidistant sulfur atoms, a [1Fe 4S] motif of eloquent simplicity. Moreover, these intricate dancers on the molecular stage were accompanied by their corresponding protein ligands—the harmonious rhythms of [3Fe 3S] in bacterial ferredoxins,^405^ and the intricate melodies of [6Fe 6S] in Desulfovibrio Vulgaris (Hildenborough).^406^

The catalytic activity of cubane-type Fe_4_S_4_ clusters in metalloproteins, such as biotin synthase,^407^ aconitase,^408^ and (E)-4-hydroxy-3-methylbut-2-enyl pyrophosphate reductase (IspH),^409^ as well as in synthetic M_4_S_4_ clusters, highlights their role in the emergence of life and the formation of organic compounds from inorganic precursors.^410^ Recent studies have revealed that Fe–S clusters with low-valent Fe^1+^ centers can adopt various electronic configurations crucial for their catalytic activity. For instance, when CO binds to a synthetic [Fe_4_S_4_]^0^ cluster, it triggers the generation of Fe^1+^ centers through intracluster electron transfer, facilitating redox reactions. Similarly, CO binding to an [Fe_4_S_4_]^+^ cluster induces electron delocalization, enabling the activation of C–O bonds without highly negative redox states.^411^ Metalloproteins containing Fe_4_S_4_ clusters can catalyze the reduction of CO and CO_2_ to hydrocarbons,^412^^,^^413^^,^^414^ which is significant in the context of Earth’s early life.

Pyruvate is a central metabolite in Archaea, Bacteria, and Eukarya, where iron-sulfur enzymes play a crucial role in connecting pyruvate to carbon fixation pathways and thioester biochemistry.^415^^,^^416^ The FeS/S/FeS_2_ system can catalyze the interconversion of hydroxyl acids and keto acids.^417^ Recent studies have shown that natural iron sulfide pyrrhotite acts as an oxidoreductase catalyst in the conversion of pyruvic acid to lactic acid^418^ and in CO_2_ reduction.^419^ However, these studies have not demonstrated the inorganic oxidoreductase activity of these nanoparticles with enzyme kinetics, contrasting with our approach in this review, which aligns with the mineral surface approach focusing on the iron-sulfur world^420^ and its relevance to evolutionary biochemistry.^421^^,^^422^

Of exceptional note, the symphony of iron sulfides, featuring a solitary iron atom coordinated with four equidistant sulfur atoms, resonated with the enthralling duet of superconductivity and high oxidoreductase activity. This symphonic revelation hinted at their potential roles within the biochemical orchestration of LUCA’s primordial milieu and the profound odyssey of the genetic code’s evolution. As we peer into this majestic tapestry woven by the interplay of matter and energy, we catch glimpses of the cosmic dance that has shaped the narrative of life itself—an unfolding tale of harmonious interactions and exquisite choreography that spans the epochs of time.

In the midst of this breathtaking panorama, whether inorganic or biological, emerges a luminous narrative—an ode to the equilibrium of chemical processes. The irresistible allure of electrons creates an invitation that transcends boundaries, beckoning toward an indomitable symphony that harmoniously merges the worlds of inorganic matter and organic life. It is a symphony that orchestrates a harmonious marriage, guiding a dance of reactivity and transformation that resonates through the annals of both chemistry and biology. Just as musical notes blend and harmonize to compose a masterpiece, the dance of electrons, photons, and Cyc-*c* mediators creates a resonant composition—a masterpiece of catalytic brilliance that elegantly unfurls on the grand stage of nature’s theater.

Recent advancements in synthesizing various inorganic nanoparticles, attributed to their intrinsic enzyme-like activity, have found wide applications in biomedicine.^25^^,^^26^^,^^27^^,^^50^^,^^51^^,^^423^^,^^424^ Fe_2_O_3_ nanocubes exhibit enhanced POD activity under visible light, enabling a photo-assisted colorimetric method for detecting glucose at low concentrations (0.1 mM detection limit).^74^ Ferrihydrite NPs, possessing CAT activity, enhance the effectiveness of radiotherapy,^49^ while magnetoferritin NPs target and visualize tumor tissues due to the iron oxide core catalyzing the oxidation of peroxidase substrates in the presence of hydrogen peroxide, producing a color reaction used to visualize tumor tissues.^425^ Additionally, dietary iron oxide NPs with CAT activity mitigate neurodegeneration in a Drosophila-Alzheimer’s disease model.^426^ The high POD and CAT activities of inorganic iron polysulfide nanoparticles are responsible for effectively inhibiting Pseudomonas aeruginosa and Staphylococcus aureus, including drug-resistant strains.^57^ These activities enable them to disrupt pathogenic biofilms, making them valuable for disinfecting implant devices such as ventilators and blood catheters. Additionally, their enzyme activity accelerates the healing of infected wounds, further extending their potential for preventing or treating biofilm-related infections.^57^ Conversely, remarkably active CeVO_4_, acting as CcO, catalyzes the four-electron reduction of dioxygen to water in the respiratory electron transport chain without releasing any partially reduced oxygen species (PROS) such as superoxide, peroxide, and hydroxyl radicals.^92^ These findings highlight the potential of iron oxide and other nanoparticles as inorganic enzymes for therapeutic and diagnostic purposes.^28^^,^^51^^,^^427^

The synthesis of inorganic iron oxide nanoparticles is an important technique in agriculture for regulating plant ROS levels in various environments.^428^^,^^429^^,^^430^ Unlike in biomedicine, where nanoparticles are typically injected into the body, plants can uptake these nanoparticles themselves.^431^ For example, inorganic iron oxide nanoparticles include those naturally occurring in soils^432^^,^^433^^,^^434^ and synthesized ones,^435^^,^^436^^,^^437^^,^^438^ which can be absorbed by roots,^435^^,^^436^^,^^437^^,^^438^^,^^439^^,^^440^^,^^441^^,^^442^ leaves,^443^ or even the surface of seeds.^444^ Once inside the plant, these nanoparticles can move to different parts of the plant, affecting various physiological processes.^445^ One notable effect of these nanoparticles is their ability to regulate ROS levels in plants, effectively acting as inorganic enzymes to manage oxidative stress,^446^^,^^447^^,^^448^ including various abiotic stresses such as drought^446^ and salt stress,^449^ as well as combating virus^443^ and fungi diseases,^450^ and reducing heavy metal contamination.^451^^,^^452^^,^^453^^,^^454^ Additionally, inorganic iron oxide nanoparticles have been found to increase the production of photosynthetic pigments in plants and electron transfer, enhancing their photosynthesis and leading to improved growth and development,^436^^,^^439^^,^^441^^,^^442^^,^^447^^,^^451^ and can even improve nitrogen fixation in soybeans.^455^ These nanoparticles can also influence plants at the molecular level, impacting gene expression and metabolic pathways,^430^^,^^431^^,^^436^^,^^453^^,^^456^ which can further affect their growth patterns. These applications demonstrate the diverse potential of inorganic nanoparticles as inorganic enzymes in addressing environmental challenges and contributing to sustainable agricultural practices.

It is worth noting that inorganic oxidoreductase is still functional in our current Earth environment. Recent results indicate that Fe_2_O_3_ nanoparticles obtained from PVC dichlorination residues and iron chips treated with subcritical water exhibit inherent peroxidase-like properties.^172^ Biogenic iron oxide nanoparticles, derived from the interaction of fungi with iron oxide nanoparticles,^170^^,^^171^ as well as magnetite nanoparticles enclosed within magnetosomes in magnetotactic bacteria,^59^^,^^60^ also demonstrate oxidoreductase activity. Furthermore, biogenic iron oxide nanoparticles from bacteria contribute to this narrative.^61^^,^^62^^,^^63^^,^^64^^,^^71^ Pyrrhotite, a natural iron sulfide mineral, has also been observed to act as an oxidoreductase in the conversion of pyruvic acid to lactic acid.^418^ It is anticipated that all iron oxide nanoparticles with the same metal structure on the current Earth will continue to function as biocatalysts, a realization yet to be fully acknowledged.

In comparison to naturally occurring iron oxide nanoparticles, the number of synthesized nanoparticles remains limited.^457^ Natural iron oxide nanoparticles are widely distributed in diverse environments, including soils, water, rocks, and living organisms^458^ (Figure S16). Extensive research and documentation on these nanoparticles have been conducted. Examples of such environments include high pH hydrothermal vents, ice sheets, fly ash, and street dust, and magnetosomes from MTB, as well as other biogenic iron minerals. These nanoparticles form through various mechanisms, resulting in different sizes, shapes, and structures. Similarly, iron sulfide nanoparticles can be found in hydrothermal vent plumes^459^^,^^460^ and many marine sediments.^461^^,^^462^^,^^463^ They have also been identified in the early Earth, predating the existence of life itself.^98^^,^^142^^,^^464^^,^^465^^,^^466^^,^^467^ Some of these nanoparticles are believed to be directly associated with the origins of life and the evolution of living organisms.^394^^,^^395^^,^^420^^,^^468^^,^^469^^,^^470^^,^^471^^,^^472^^,^^473^^,^^474^

It is important to note that not all nanoparticles exhibit biocatalytic properties. The activity of inorganic enzymes is directly linked to their metal structure, which can be influenced by both biological and environmental conditions. Therefore, further research is needed to elucidate the nuanced relationship between the structure of inorganic enzymes and their catalytic activity in different environmental contexts. Additionally, the role of inorganic enzymes in ecosystems, particularly in the pre-protein world, remains an understudied area that holds great potential for uncovering fundamental aspects of biochemical evolution and the origin of life. Future studies focusing on the function of natural inorganic nanoparticles or inorganic nanocolloids are necessary to deepen our understanding of their significance in biological and environmental systems, including addressing the challenge of ROS in water eutrophication and toxicity, air pollution, and global climate change. This underscores the importance of studying natural nanoparticles and their functions, which are currently underexplored.

Furthermore, the applications of inorganic nanoparticles, especially those synthesized, are not limited to current biomedicine, agriculture, and environmental assessment. They can also be further extended to address various challenges in our lives related to global climate change, water quality, and sustainable development. Research in these areas could lead to innovative solutions that leverage the unique properties of inorganic nanoparticles to tackle pressing global issues.

## Conclusion

In summary, the research illuminates the extraordinary oxidoreductase activity displayed by inorganic iron oxide and sulfide nanoparticles. This activity is driven by their distinctive metal architecture and electron conductivity. These characteristics set the stage for electron hopping, a phenomenon vital for disrupting chemical equilibrium and initiating reactions. Additionally, the involvement of light activation and Cyc *c* electron mediators further amplifies their catalytic potential.

These inorganic nanoparticles, akin to fascinating “life fossil oxidoreductases," provide a unique window into the origins and development of life on Earth. They represent one of the earliest classes of oxidoreductases known as inorganic enzymes, predating the emergence of more complex biological molecules. They represent one of the earliest-known classes of oxidoreductases, predating the emergence of more complex biological molecules. In ancient environments characterized by hydrogen peroxide and free radicals, these nanoparticles played a pivotal role in fundamental biochemical processes, including redox reactions, detoxification, and nutrient cycling.

Today, inorganic iron oxide and sulfide nanoparticles continue to serve as essential biocatalysts in natural ecosystems. Found in soils, sediments, and aquatic environments, they mediate crucial reactions that contribute to the overall health and functioning of ecosystems. Their enduring impact on Earth’s history underscores their significance, shaping the delicate symphony of our planet’s ecosystems.

The electron transfer property stands as a vital bridge between inorganic nanoparticles and proteins, particularly in the context of metal centers and their evolution. This property is a fundamental aspect that unites their catalytic capabilities. The coordination of metal centers within proteins is resonant with the metal architecture of inorganic nanoparticles, and both contribute significantly to the electron transfer dynamics essential for various biochemical processes.

As we delve deeper into the world of inorganic enzymes, exploring their electron transfer capabilities, electron hopping phenomena, and their responsiveness to light and Cyc *c* mediation, we gain invaluable insights into the fundamental principles that governed early life processes and adaptations. These nanoparticles continue to be pivotal players in the ongoing dance of life on our planet, leaving an indelible mark on Earth’s intricate web of existence.

Moving forward, while current studies are more focused on the biomedical applications of synthesized nanoparticles, it is important to broaden the scope to include the exploration of natural nanoparticles and their role in ecosystem function. Understanding the characteristics of enzyme activity exhibited by both natural and synthesized nanoparticles is crucial for comprehensively assessing their potential impact. It is important to emphasize that the activity of nanoparticles is inherent to their nanoparticle physics and is not a product of deliberate design by scientists. This activity has existed even before the protein world and should be researched and applied in a broader context, encompassing environmental challenges and sustainable development.

The scope of this field should not be limited to synthesized nanoparticles with biomedical applications. Understanding the role of natural inorganic nanoparticles as inorganic enzymes in our current ecosystem is crucial for addressing environmental challenges, including their impact on plant growth and water purification mechanisms (e.g., eutrophication and chemical detoxification), as well as their interactions with ROS in the atmosphere and air pollution contributing to global climate change. Such studies can broaden our understanding of the significance of these nanoparticles in biogeochemistry and their potential applications in diverse fields, including climate science, atmospheric chemistry, soil science, agriculture, and environmental management.

Furthermore, studying the characteristics and activities of nanoparticles provides an opportunity to gain insights into the origin of life and evolution. By understanding how nanoparticles interacted with early Earth environments and potentially played a role in the emergence of life, we can gain a deeper understanding of the processes that led to the development of life on our planet. This broader perspective can provide valuable insights into the fundamental principles of biology and evolution.

This study highlights the pivotal role of inorganic enzymes in bridging ancient and modern biology, illustrating their significance from ecosystem to evolution in both chemistry and biology. The hypothesis on inorganic enzymes stemmed from the author’s observation of phosphate ester behavior in artificial seawater in 2006,^30^ triggered by Graham Cairns-Smith’s book “Seven Clues to the Origin of Life.”^475^ I dedicate this work to my wife, Wei Sun, and my son, Jack Jixiang Huang, for their unwavering support and sacrifices throughout my academic journey, especially during the COVID-19 pandemic when I worked independently from home. My inspiration was ignited by J.D. Bernal’s seminal work “The Physical Basis of Life”^476^ and by the persistent questioning of Dr. Michael J. Russell regarding the energy for these intricate systems. I also acknowledge the guidance and encouragement of Drs. Gerhard Schenk and the late R.J.P. Williams, as well as the support of Drs. Robert Atlas, Jia-Zhong Zhang, and Peter B. Ortneras, for their instrumental contributions to the development of my research.
