# Supplementary material for: Unveiling the role of inorganic nanoparticles in Earth’s biochemical evolution through electron transfer dynamics
Source: iScience. 2024 Mar 25;27(5):109555. doi: 10.1016/j.isci.2024.109555 (PMC11024932; doi:10.1016/j.isci.2024.109555)
Supplement: Document S1. Figure S1–S16 [file mmc1.pdf]

iScience, Volume *27*

Supplemental information

Unveiling the role of inorganic nanoparticles in Earth's biochemical evolution through electron transfer dynamics

Xiao-Lan Huang

SUPPORT INFORMATION

Figure captions

Fig. 1 Characteristics of inorganic phosphatase.

A. The enzyme-like activity of inorganic iron oxide nanoparticles solution using the dialysis membrane tube (DMT), Lineweaver–Burk plot of three organic phosphate compounds (Glycerol-2-phosphate G2P, glucose-6-phosphate G6P, and Adenosine triphosphate ATP) and two condensed phosphates (Polyphosphate Poly-Pi. and Pyrophosphate PPi) (Reprinted from Ref. [1,2])
B. The inhibiting behavior of different tetrahedral oxyanions on the hydrolysis of G6P in a 10 month aged, 1000 nM $Fe(NO_3)_3$ solution at room temperature (22 °C) (Reprinted from Ref[3,4]).
C. The effect of temperature on the hydrolysis velocity of ATP (Reprinted from Ref. [1])
D. Effect of pH on the hydrolysis velocity of ATP of ATP (Reprinted from Ref. [1])

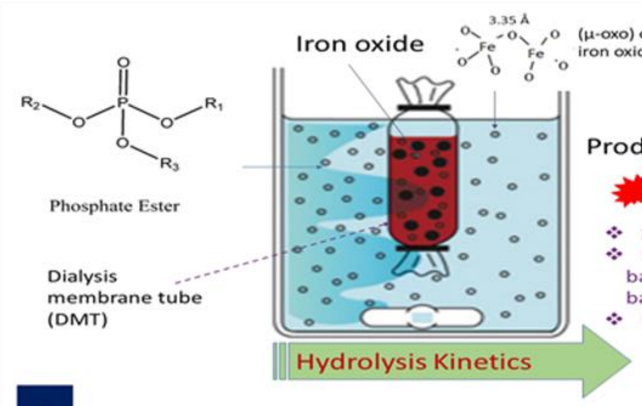

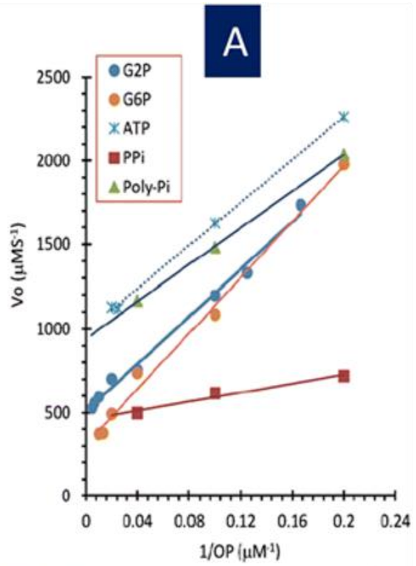

B ❑ Inhibiting behaviour

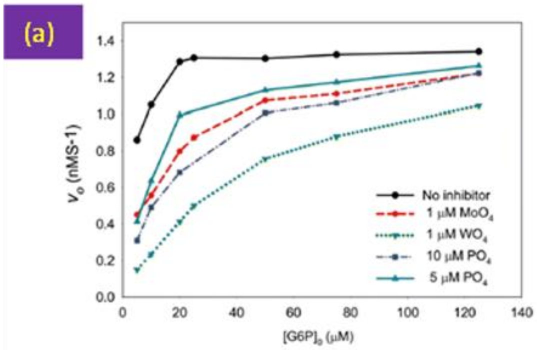

Michaelis-Menten constant (K_m) and maximum velocity (V_m) of different phosphorus in a DMT-inorganic oxide solution.

P source	V_m (nM S^{-1})	K_m (μM)	Range (μM)	r^2
G2P	2.0	7.0	6 to 200	0.99
G6P	3.2	8.3	5 to 100	0.99
ATP	0.9	9.2	5 to 50	0.99
poly-P_i	1.1	5.5	5 to 25	1.00
PP_i	2.2	1.3	5 to 25	0.98

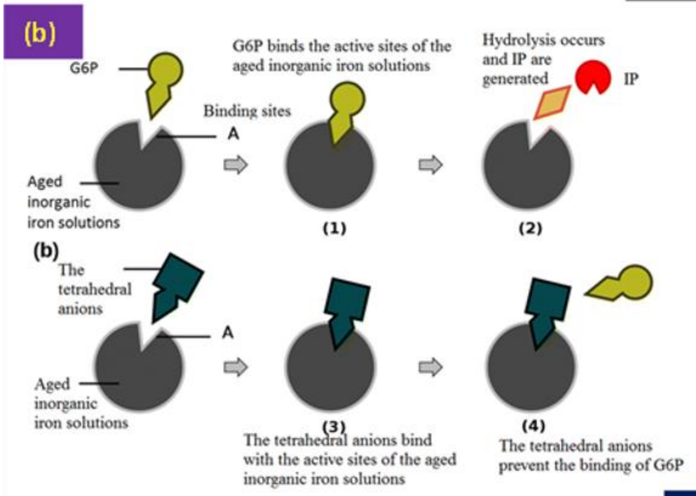

$$E + S \underset{k_{-1}}{\overset{k_1}{\rightleftharpoons}} ES \xrightarrow{k_2} E + P$$

$$+I \downarrow\uparrow -I \qquad K_i = \frac{[E][I]}{[EI]}$$

$$EI \not\rightarrow \text{(N.R.)}$$

Diagram of catalysis process of G6P hydrolysis in the presence of the tetrahedral anions in a 10-month aged 1000 nM $Fe(NO_3)_3$ solution.containing iron oxide nanoparticles

❑ Temperature

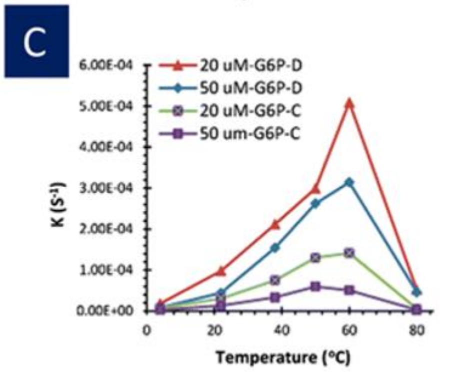

❑ pH

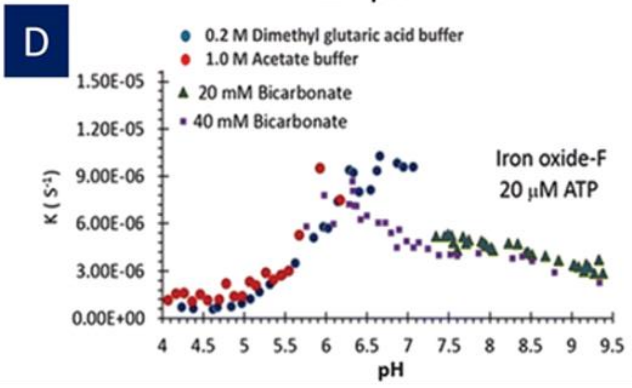

Fig. 2 Catalase Activity of Inorganic Iron Oxide Nanoparticles[5]

A. SEM images of 10 different iron oxide nanomaterials synthesized using the method of Cornell and Schwertmann[6], including 2-line ferrihydrite (2L-Fht, $Fe_5HO_8 \cdot 4H_2O$), 6-line ferrihydrite (6L-Fht, $Fe_5HO_8 \cdot 4H_2O$), goethite (Goe, α-FeOOH), akageneite (Aka, β-FeOOH), lepidocrocite (Lep, γ-FeOOH), feroxyhyte (Foh, δ′-FeOOH), hematite (Hem, α-Fe_2O_3), maghemite (Mah, γ-Fe_2O_3), magnetite (Mag, Fe_3O_4) and schwertmannite (Sch, $Fe_8O_8(OH)_6SO_4$). Scale bar, 200 nm.

B. The formation of dissolved O_2 in 100 mM H_2O_2 solution containing various iron oxide nanomaterials (10 µg/mL), measured by using a specific oxygen electrode on a multi-parameter analyzer (JPSJ-606L, Leici China). The total volume of the mixture was 5 mL. All reactions were carried out in deoxygenated water at 37°C.

C. The number of hydroxyl groups on the surface of various iron oxide nanomaterials measured by acid-base titration[7] is positively correlated with their corresponding catalase-like activity (expressed by the rate of dissolved oxygen generation).

D. Schematic illustration of inorganic catalase activity *vs* surface hydroxyl group of iron oxide NPs

Reprinted from Ref [5]

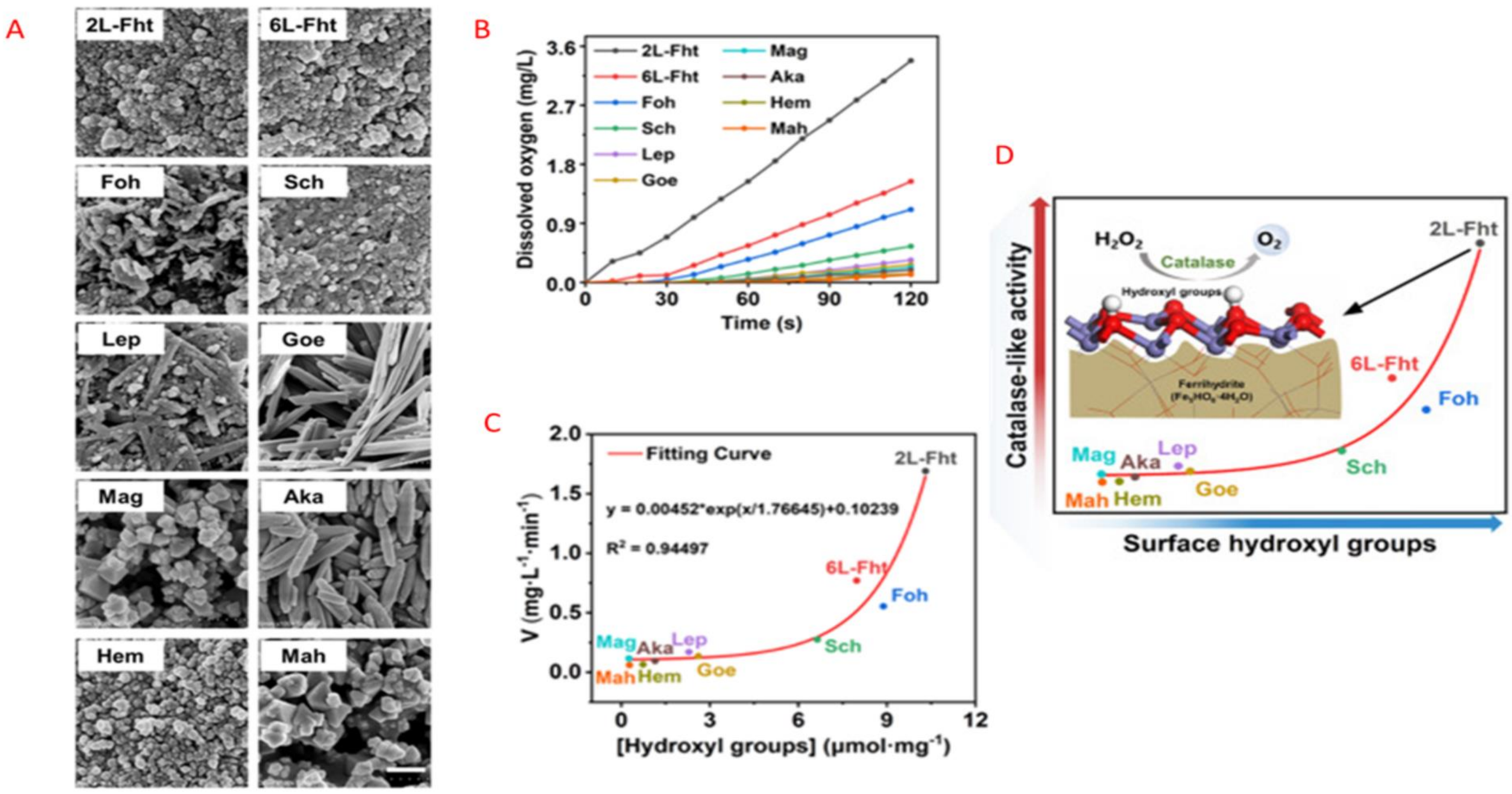
A
2L-Fht
6L-Fht
Foh
Sch
Lep
Goe
Mag
Aka
Hem
Mah
B
Dissolved oxygen (mg/L)
Time (s)
2L-Fht
6L-Fht
Foh
Sch
Lep
Goe
Mag
Aka
Hem
Mah
C
Fitting Curve
y = 0.00452*exp(x/1.76645)+0.10239
R^2 = 0.94497
V ($mg \cdot L^{-1} \cdot min^{-1}$)
[Hydroxyl groups] ($\mu mol \cdot mg^{-1}$)
D
H_2O_2
Catalase
O_2
Hydroxyl groups
Ferrihydrite
Catalase-like activity
Surface hydroxyl groups

Fig 3. **Intrinsic Oxidoreductase Activity of Various Iron Sulfites Nanoparticles**

A. Color change of suspensions of sheet-like FeS to show the peroxidase activity. (1) Images of the suspension of sheet-like FeS nanostructure, (2) mixture of TMB and H_2O_2 after catalytic reaction by sheet-like FeS nanostructure, and (3), mixture of TMB and H_2O_2 after adding H_2SO_4 to quench the catalytic reaction by sheet-like FeS nanostructure. Reprinted with permission from Ref. [8], Copyright © 2009, John Wiley & Sons

B. Peroxidase activity of various iron sulfides nanoparticles (a) SEM and TEM images of Cys-nFeS from different amount of cysteine. Fe_3O_4 represents no cysteine in the reaction. Scale bars in SEM images: 5 μm. Scale bars in TEM images: 200 nm. Representative images are shown. (b). Michaelis-Menten kinetics of peroxidase-like activity of Cys-nFeS (with varied H_2O_2). (c) The trend of *Km* and the ratio of V_{max}/K_m in the kinetics assay with varied H_2O_2. (d) Michaelis-Menten kinetics of peroxidase-like activity of Cys-nFeS (with varied TMB). (e) The trend of K_m and the ratio of $Vmax/K_m$ in the kinetics assay with varied TMB, respectively. Reprinted from Ref. [9].

C. Catalase-like activity of Cys-nFeS. (a) The catalase-like activity to decompose H_2O_2 into oxygen (bubble) by Cys-nFeS.Representative images are shown. (b) The trend of *Km* and the ratio of V_{max}/Km in the kinetics assay with varied cysteine. (C) The trend of KM and the ratio of V_{max}/Km in the kinetics assay with varied H_2O_2. Reprinted from Ref. [9].

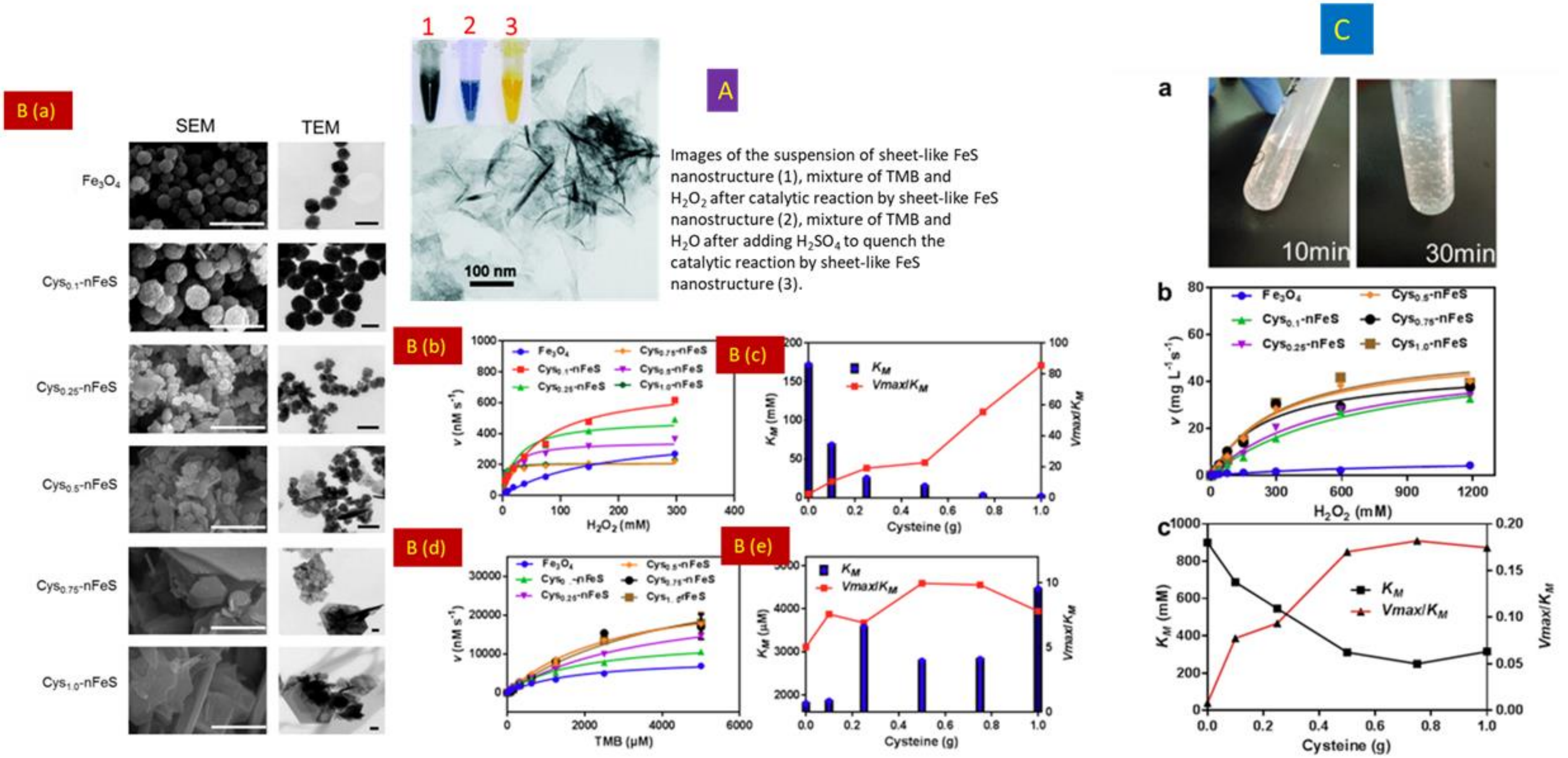

Images of the suspension of sheet-like FeS nanostructure (1), mixture of TMB and H_2O_2 after catalytic reaction by sheet-like FeS nanostructure (2), mixture of TMB and H_2O after adding H_2SO_4 to quench the catalytic reaction by sheet-like FeS nanostructure (3).

Fig 4. Metal Architecture of Inorganic Nanomaterials and POD-like Activity (by DFT modelling)[10]

A. Proposed mechanism of the POD-like activity of nanomaterials in three steps. Eb,1, Eb,2, and Eb,3 are the energy barriers; Er,1, Er,2, and Er,3 are the corresponding reaction energies.

B. Diagrams of iron-oxide slabs with different crystal structures, exposed facets, defects, and chemical modifications (1–15). The top surfaces were used for the catalysis. FeO, Fe_3O_4, and Fe_2O_3 are ferrous, ferriferous, and ferric oxides. For slab number 2, two O atoms on top; 4, one O atom on top; 5, two hydroxyl groups on top; 6, one O vacancy on top; 11, two Fe vacancies on top; 12, one Fe vacancy on the subsurface; 13, four Fe vacancies on top; 14, two Fe vacancies on the subsurface; 15, two hydroxyl groups and four Fe vacancies on top.

C. Structures of the other NMs with different crystal structures, e.g. monometallic oxides (TiO_2, V_2O_5, Mn_3O_4, Fe_3O_4, Co_3O_4, NiO, Cu_2O, ZrO_2, and RuO_2) and perovskites ($LaNiO_3$, $LaCoO_3$, $LaMnO_3$, and $SrFeO_3$).

D. Volcano-shaped activity curve. The colour change from blue to white represents the gradual reduction in the activity of POD-like activity from a high value to zero. The labels 1–15 represented the corresponding iron oxide structure in (B).

E. Overview of POD-like activities of different nanomaterials, corresponding C. The blue zone is the POD activity window defined by the hydroxyl adsorption energy (Eads, OH).

Reprinted from Ref. [10]

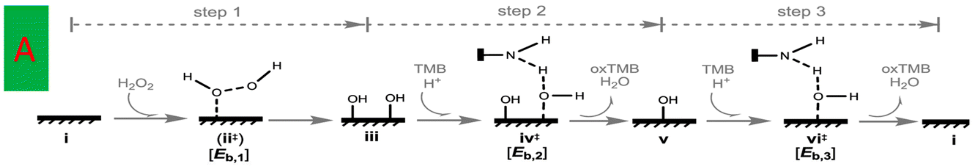

$$H_2O_2 \xrightarrow{E_{b,1}} 2OH^*,\ E_{r,1} \quad (1)$$

$$OH_1^* + TMB + H^+ \xrightarrow{E_{b,2}} oxTMB + H_2O,\ E_{r,2} \quad (2)$$

$$OH_2^* + TMB + H^+ \xrightarrow{E_{b,3}} oxTMB + H_2O,\ E_{r,3} \quad (3)$$

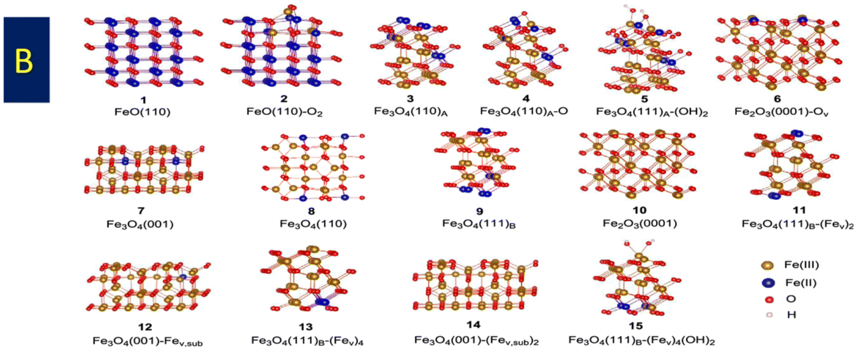

The mechanism involves chemisorption of H2O2 onto the surface to form two hydroxyl adsorbates and two subsequent reduction processes to remove the hydroxyl groups from the surface. Eb,1, Eb,2, and Eb,3 are the energy barriers; and Er,1, Er,2, and Er,3 are the corresponding reaction energies.

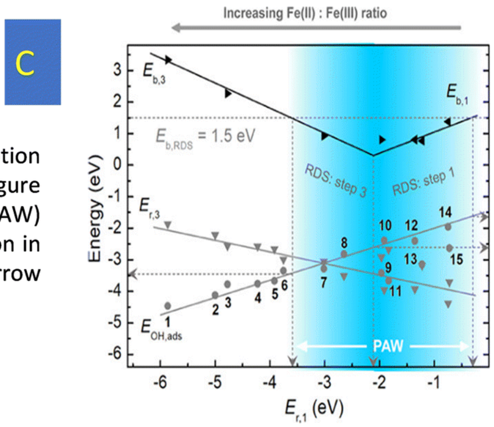

The volcano-shaped plot of POD activity (black lines) as a function of H2O2 chemisorption energy (Er,1) for iron-oxide slabs 1−15 (b) Er,3, and Eads,OH are also plotted in the figure as linear functions of Er,1 (gray lines). The blue zone is the POD activity window (PAW) defined by Er,1. The color change from blue to white represents the gradual reduction in the activity from a high value to zero. The vertical and horizontal dashed lines with arrow illustrate how the PAWs are defined by Er,1 and Eads,OH, respectively.
Labels ●, ▼, ◀, and ▶ designate Eads,OH, Er,3, Eb,1, and Eb,3, respectively.

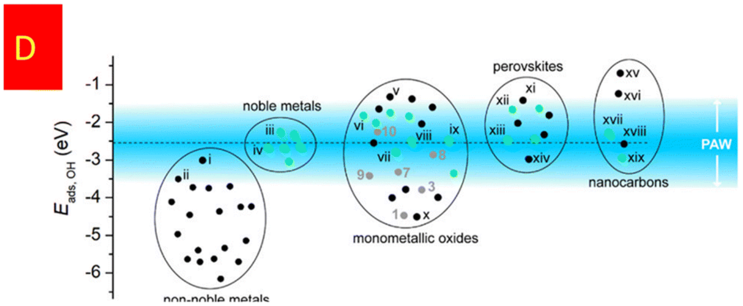

The blue zone is the PAW defined by the hydroxyl adsorption energy (Eads,OH). The color change from blue to white represents the gradual reduction in the activity from a high value to zero. The green-filled circles represent POD-mimetic nanozymes already available in the literature, and the gray-filled circles represent the iron-oxide nanosurfaces studied in this work.

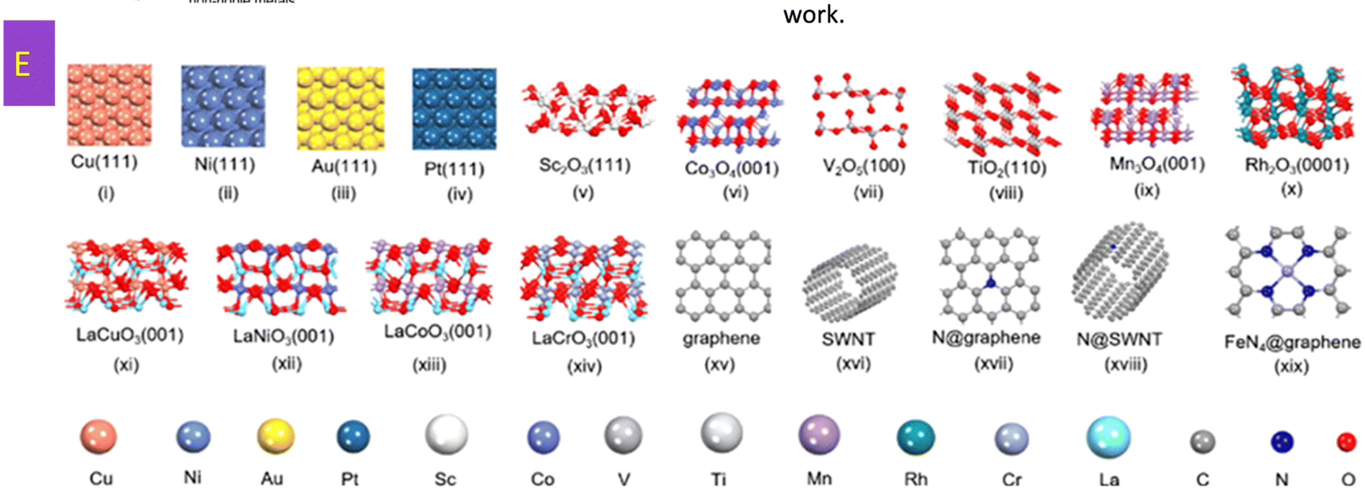

Fig 5. Intrinsic Oxidoreductase Activity of Biogenic Iron Oxide Nanoparticles

A. The intrinsic CAT-like activity of BMNPs from Burkholderia sp. YN01.Strain YN 01. (a) Time-course curve of H2O2 decomposition by BMNPs as a catalase mimic. (b) B. Effect of BMNP concentration on catalytic activity. Reaction conditions: 30 μg ml^{-1} BMNPs or a series of concentrations, 2.5 mmol l^{-1} H_2O_2 and 200 mmol l^{-1} Na_2HPO_4-citric acid buffer (pH 9), or 0.5 U ml^{-1} catalase, 5 mmol l^{-1} H_2O_2 and 200 mmol l^{-1} Na_2HPO_4-citric acid buffer (pH 8). Reprinted with permission from Ref. [11], Copyright © 2019, John Wiley & Sons

B. POD activity from Ferritin Core, (a) TEM images of ferritin samples and the analysis of its peroxidase-like activity using TMB, DPD, and OPD as substrates at 50 °C. (b) Analysis of peroxidase-like activity of ferritin using p-HPPA (left) and luminol (right) as substrates, (c) The comparison of thermal stability (left) and pH tolerance (right) of peroxidase activity between ferritin and HRP Reprinted with permission from Ref. [12], Copyright © 2011, ACS

C. SOD activity from Ferritin Core, from archaea (P. furiosus and P. yayanosii, pfFn & pyFn, S. solfataricus, ssDps), bacteria (E. coli, EcDps, DNA-binding proteins from starved cells, ferritin (FTn) from bacteria (EcFTn), and bacterioferritin from bacteria (EcBfr), and eukaryotes (H. sapiens, heavy chain ferritin (HFn) & light chain ferritin (LFn). The inhibition rate, indicative of SOD-like activity, of biomineralized ferritins with a final protein concentration of 50 μg/mL, presented as the mean ± SD (n = 3 independent experiments). Reprinted from Ref[13]

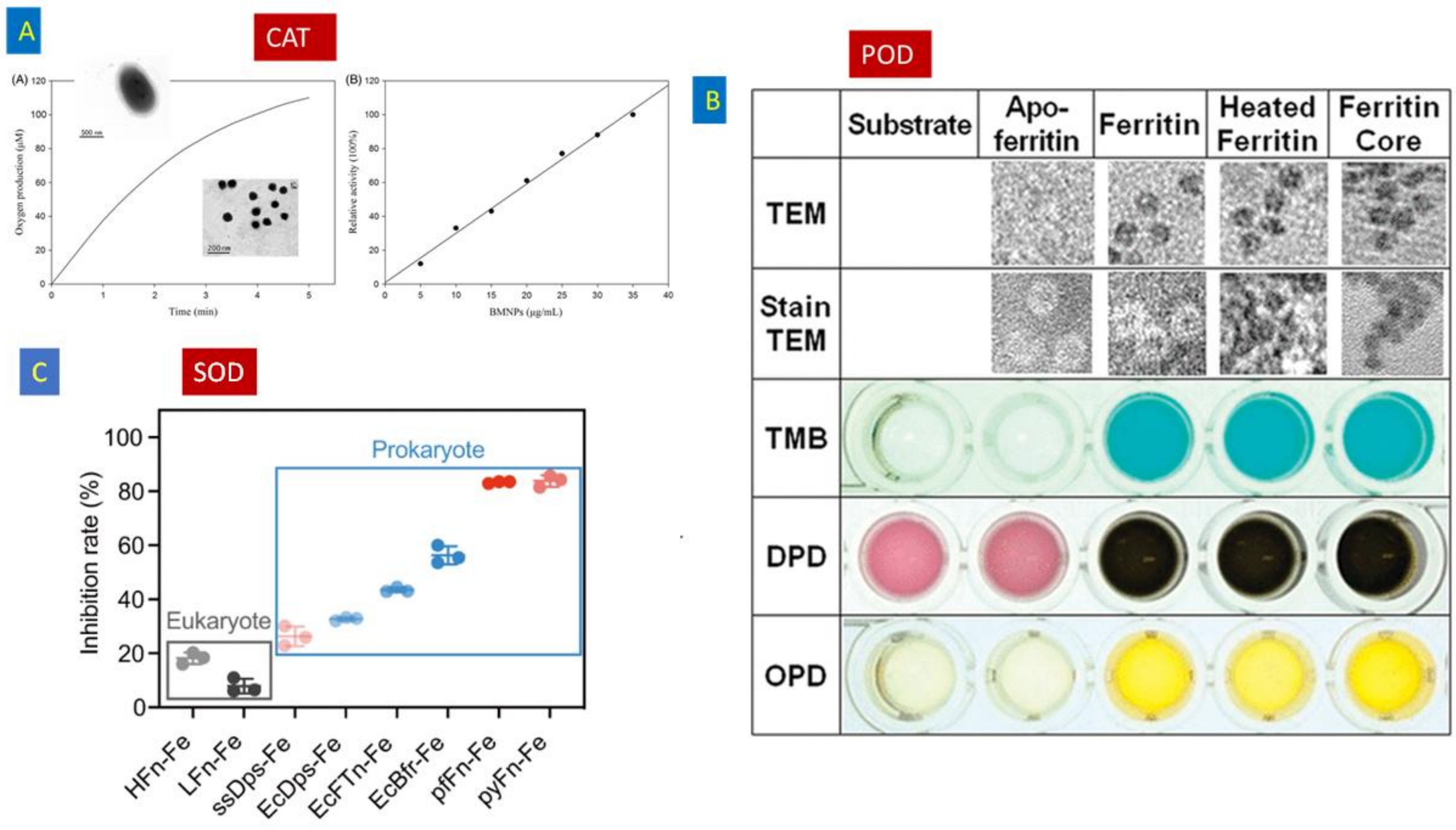
A
CAT
(A)
Oxygen production (μM)
Time (min)
500 nm
200 nm
(B)
Relative activity (100%)
BMNPs (μg/mL)
C
SOD
Inhibition rate (%)
Eukaryote
Prokaryote
HFn-Fe
LFn-Fe
ssDps-Fe
EcDps-Fe
EcFTn-Fe
EcBfr-Fe
pfFn-Fe
pyFn-Fe
B
POD
Substrate
Apo-ferritin
Ferritin
Heated Ferritin
Ferritin Core
TEM
Stain TEM
TMB
DPD
OPD

Fig. 6. POD Activity of CuO Nanorods (CuO NRs) Under UV–visible Light[14].

A. UV–visible absorbance spectra of ABTS oxidation under different conditions: (i) ABTS + H_2O_2; (ii) CuO NRs + ABTS; (iii) CuO NRs + ABTS + H_2O_2.
B. UV–visible absorbance spectra of different peroxidase substrates using CuO NRs in the presence of H_2O_2: (i) OPD; (ii) TMB; (iii) ABTS. Insets show the color of postreaction solutions.
C. Double-reciprocal Lineweaver–Burk plots of the catalytic activity of CuO NRs at a fixed concentration of H_2O_2 versus varying concentrations of the ABTS. The red and blue fittings represent the experiments conducted without and with visible light illumination, respectively.
D. Double-reciprocal Lineweaver–Burk plots of the catalytic activity of CuO NRs at a fixed concentration of ABTS versus varying concentrations of H_2O_2. The red and blue fittings represent the experiments conducted without and with visible light illumination, respectively.
E. Suggested Mechanism of CuO NRs promoted by UV-visible light. The favorable energy-band structure of CuO NRs allows them to spontaneously produce •OH radicals.

Reprinted with permission from Ref. [14]. Copyright © 2018 ACS

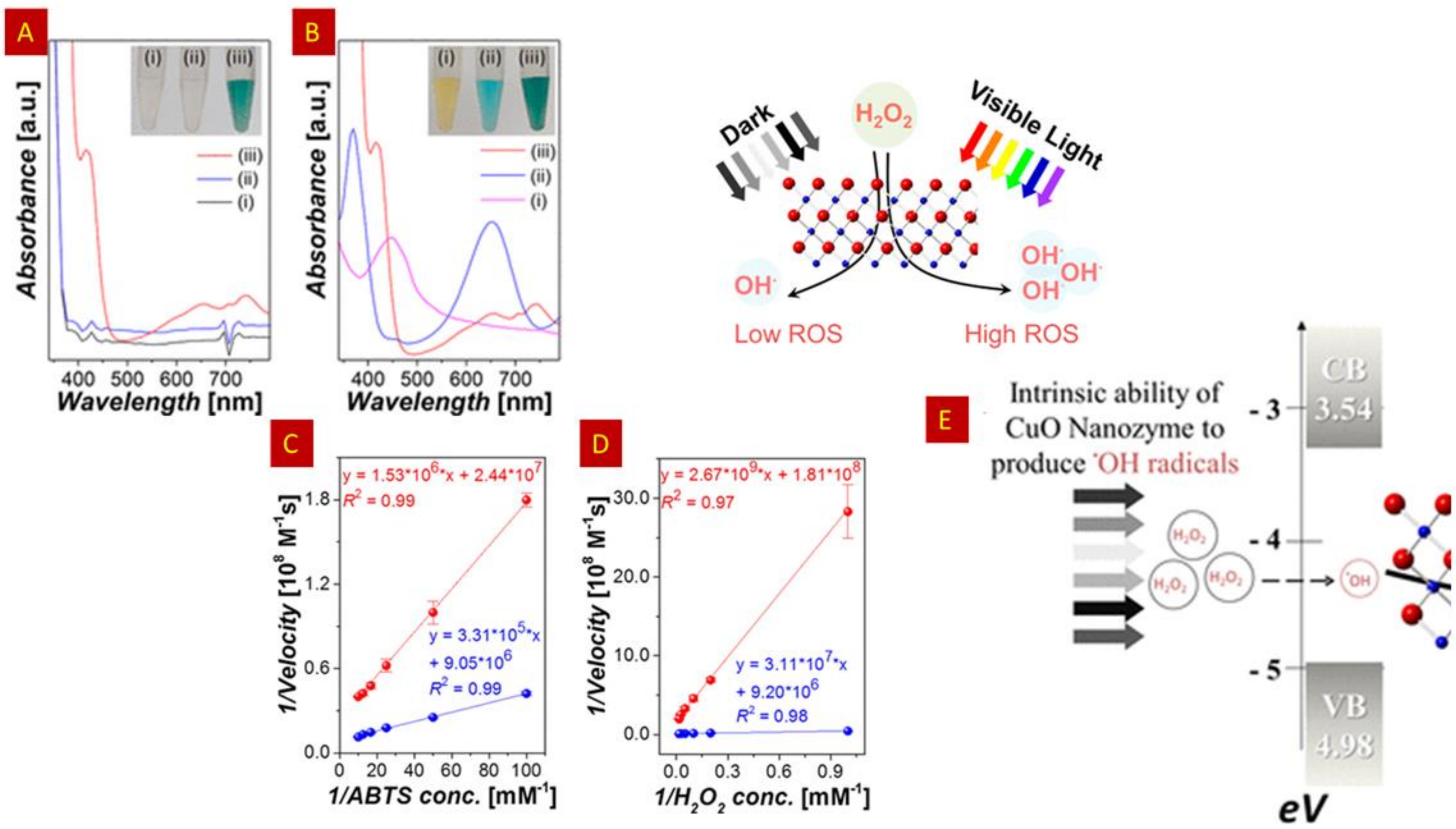
A
Absorbance [a.u.]
Wavelength [nm]
(i) (ii) (iii)
B
Absorbance [a.u.]
Wavelength [nm]
(i) (ii) (iii)
Dark
Visible Light
H2O2
OH·
Low ROS
High ROS
C
1/Velocity [10^8 M^-1s]
1/ABTS conc. [mM^-1]
y = 1.53*10^6*x + 2.44*10^7
R^2 = 0.99
y = 3.31*10^5*x
+ 9.05*10^6
R^2 = 0.99
D
1/Velocity [10^8 M^-1s]
1/H2O2 conc. [mM^-1]
y = 2.67*10^9*x + 1.81*10^8
R^2 = 0.97
y = 3.11*10^7*x
+ 9.20*10^6
R^2 = 0.98
E
Intrinsic ability of
CuO Nanozyme to
produce ·OH radicals
CB
3.54
VB
4.98
eV

Fig. 7: Metal Architecture of Iron Oxide

A. Main structure types of iron oxides[15] Reprinted with permission from Dr Jean-Pierre Jolivet.

B. The Baker-Figgis d-Keggin cluster Fe_{13}[16]. Polyhedral representation of the ideal ferrihydrite structure viewed along the c axis. The central FeO_4 tetrahedra are surrounded by 12 FeO_6 octahedra. Reprinted with permission from Ref[16], Copyright © 2007, AAAS

C. The basic structural motif consist of a central FeO_4 tetrahedra with 12 FeO_6 octahedra. The hexagonal unit cell for Ferrihydrite, Fe-oxo-Fe bridge at the edge-shared octahedra[16]. The The bonded atoms (yellow) define a cubane-like moiety that connects the basic structural motif of the model.

D. The Fe-Fe distance and linkage of octahedra in Fe(III) oxides[6,17] Reprinted with permission from Ref [6], Copyright © 2003, John Wiley and Sons

E. Crystal structures of magnetite, maghemite and hematite, Reproduced from Ref. [18] with permission from the Royal Society of Chemistry(RSC).

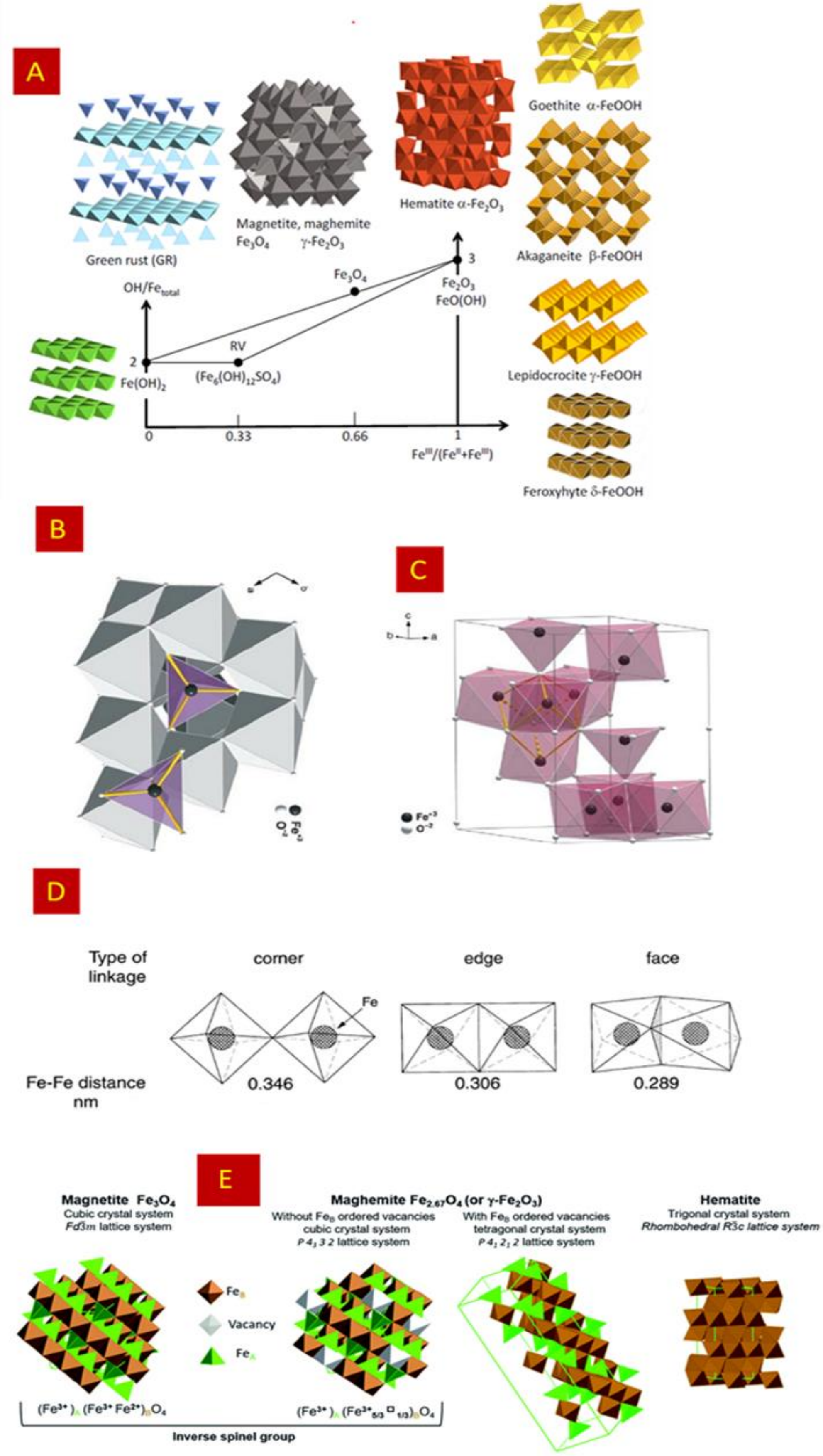

A
Green rust (GR)
Magnetite, maghemite
Fe3O4 γ-Fe2O3
Hematite α-Fe2O3
Goethite α-FeOOH
Akaganeite β-FeOOH
Lepidocrocite γ-FeOOH
Feroxyhyte δ-FeOOH
OH/Fetotal
Fe3O4
3
Fe2O3
FeO(OH)
RV
2
Fe(OH)2
(Fe6(OH)12SO4)
0
0.33
0.66
1
FeIII/(FeII+FeIII)
B
Fe+3
O-2
C
Fe+3
O-2
D
Type of linkage
corner
edge
face
Fe
Fe-Fe distance nm
0.346
0.306
0.289
E
Magnetite Fe3O4
Cubic crystal system
Fd3m lattice system
Maghemite Fe2.67O4 (or γ-Fe2O3)
Without FeB ordered vacancies
cubic crystal system
P 43 3 2 lattice system
With FeB ordered vacancies
tetragonal crystal system
P 41 21 2 lattice system
Hematite
Trigonal crystal system
Rhombohedral R3c lattice system
FeB
Vacancy
FeA
(Fe3+)A (Fe3+ Fe2+)B O4
(Fe3+)A (Fe3+5/3 □1/3)B O4
Inverse spinel group

Fig 8: Dynamic Metal Architecture of Iron Oxide Nanoparticles in the Environment

- **A.** Main pathways for the formation of magnetite (Fe_3O_4) and green rust (GR)[19], including the abiotic and biotic processing . Reprinted with permission from Ref[19], Copyright © 2007, ACS
- **B.** Magnetite NPs formation from the ferrihydrite (F), lepidocrocite (L), and goethite (G) NPs in the aged solution with the supply of ferrous ion [20] Reprinted with permission from Ref[20],Copyright © 2012, Elsevier
- **C.** Interaction of solar radition Reprinted with permission from Ref[21], Copyright © 2019, ACS.

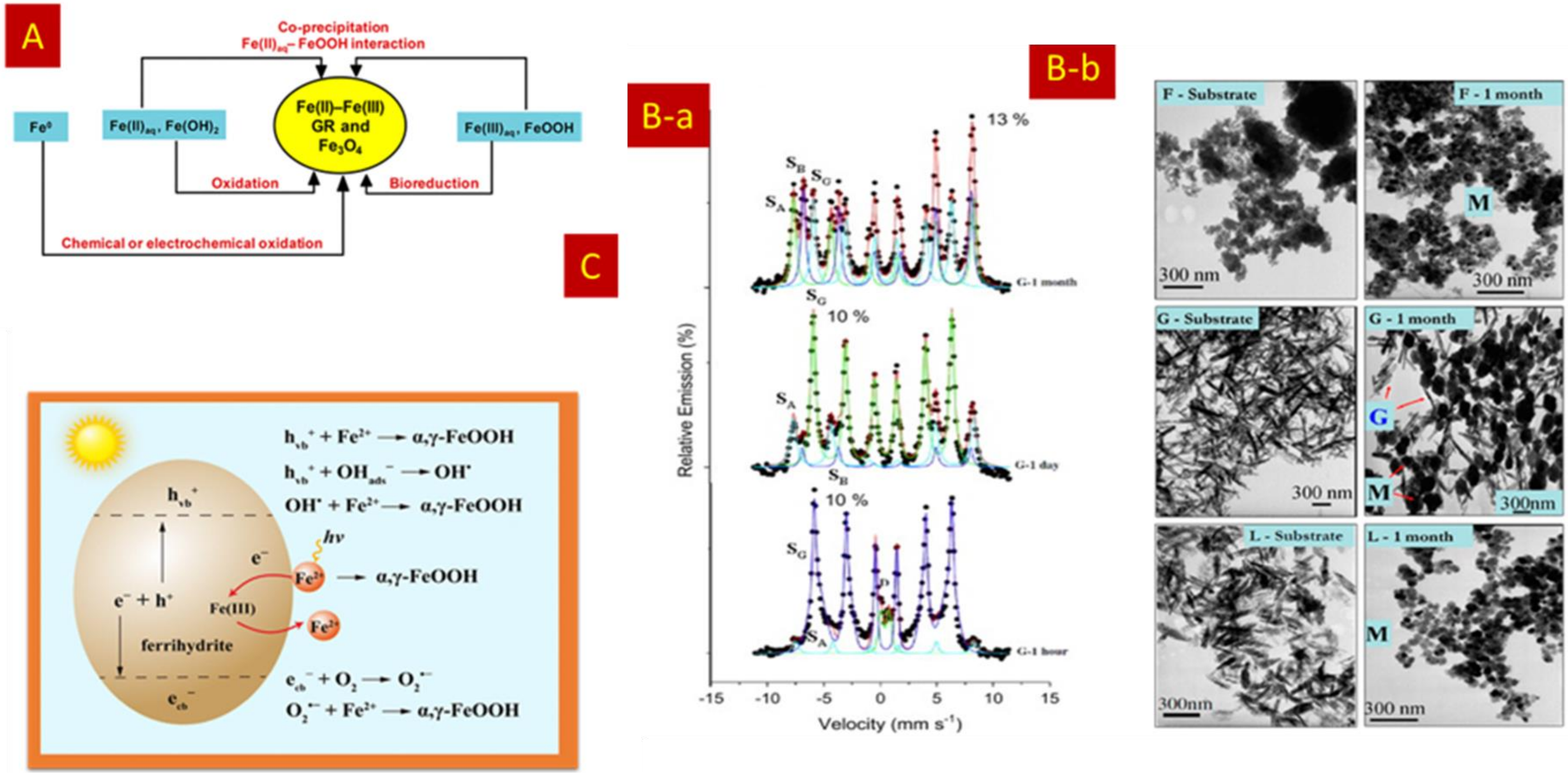
A
Co-precipitation
Fe(II)aq– FeOOH interaction
Fe0
Fe(II)aq, Fe(OH)2
Fe(II)–Fe(III) GR and Fe3O4
Fe(III)aq, FeOOH
Oxidation
Bioreduction
Chemical or electrochemical oxidation
C
hvb+ + Fe2+ → α,γ-FeOOH
hvb+ + OHads− → OH•
OH• + Fe2+ → α,γ-FeOOH
hv
e−
Fe2+ → α,γ-FeOOH
e− + h+
Fe(III)
Fe2+
ferrihydrite
ecb− + O2 → O2•−
O2•− + Fe2+ → α,γ-FeOOH
hvb+
ecb−
B-a
B-b
Relative Emission (%)
Velocity (mm s−1)
13 %
10 %
10 %
SA
SB
SG
G-1 month
G-1 day
G-1 hour
F - Substrate
F - 1 month
G - Substrate
G - 1 month
L - Substrate
L - 1 month
M
G
300 nm

Fig 9. Structure Change of Magnetite Nanoparticles in the POD Catalytic Processing [22]

A. The specific activity (a_{nano}) of these three IONPs with TMB as colorimetric substrates.
B. Kinetic study of a_{nano} values of Fe_3O_4 NPs with the days of cyclic catalytic reaction. Error bars represent standard deviation from three independent measurements.
C. Comparison of Fe L_2, and L_3 spectra of Fe_3O_4 NPs before and after 5 days of cyclic POD-like reactions
D. The fitted Fe2p XPS spectra of Fe_3O_4 NPs recycled after catalysis on days 0, 1, 3, and 5.
E. The Fe L-edge NEXAFS spectra of Fe_3O_4 NPs and recycled Fe_3O_4 NPs after 5 days of catalysis in comparison with the reference spectra of $FeSO_4$ and Fe_2O_3.
F. Raman spectra of Fe_3O_4 NPs recycled after catalysis on days 0, 1, 3, and 5.
G. TEM, HRTEM images, and SAED pattern of Fe_3O_4 NPs and recycled Fe_3O_4 NPs after 5 days of catalysis. Images were collected at least three times for each type of NPs
H. Schematic diagram of the catalytic mechanism of the POD-like activity for Fe_3O_4 NPs.

Reprinted from Ref. [22]

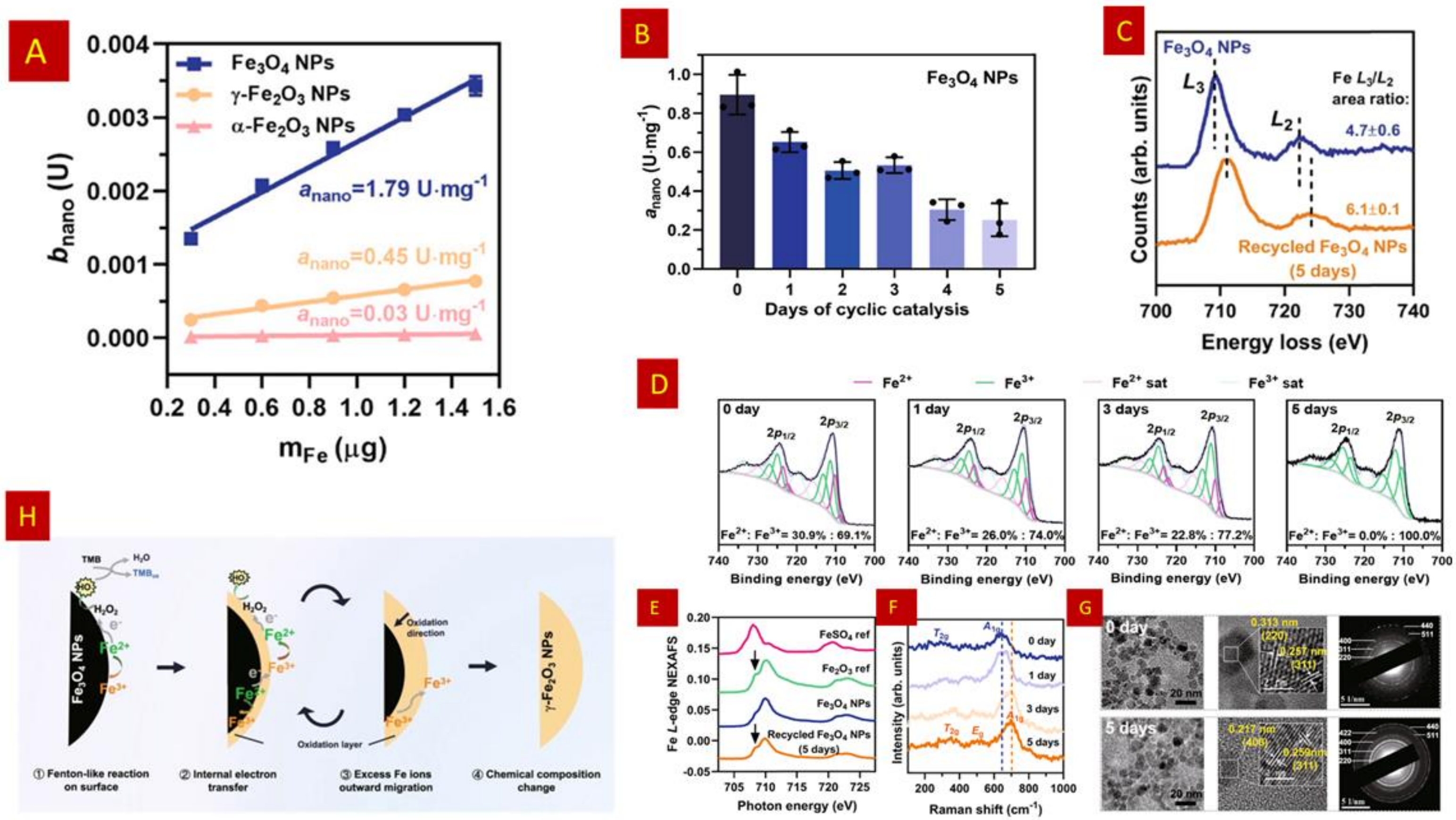
A
Fe3O4 NPs
γ-Fe2O3 NPs
α-Fe2O3 NPs
anano=1.79 U·mg-1
anano=0.45 U·mg-1
anano=0.03 U·mg-1
bnano (U)
mFe (μg)
B
Fe3O4 NPs
anano (U·mg-1)
Days of cyclic catalysis
C
Fe3O4 NPs
L3
L2
Fe L3/L2 area ratio:
4.7±0.6
6.1±0.1
Recycled Fe3O4 NPs (5 days)
Counts (arb. units)
Energy loss (eV)
D
Fe2+
Fe3+
Fe2+ sat
Fe3+ sat
0 day
1 day
3 days
5 days
2p1/2
2p3/2
Fe2+: Fe3+= 30.9% : 69.1%
Fe2+: Fe3+= 26.0% : 74.0%
Fe2+: Fe3+= 22.8% : 77.2%
Fe2+: Fe3+= 0.0% : 100.0%
Binding energy (eV)
E
FeSO4 ref
Fe2O3 ref
Fe3O4 NPs
Recycled Fe3O4 NPs (5 days)
Fe L-edge NEXAFS
Photon energy (eV)
F
T2g
A1g
Eg
0 day
1 day
3 days
5 days
Intensity (arb. units)
Raman shift (cm-1)
G
0 day
5 days
20 nm
0.313 nm (220)
0.257 nm (311)
0.217 nm (400)
0.259 nm (311)
H
TMB
H2O
H2O2
Fe3O4 NPs
Fe2+
Fe3+
Oxidation direction
Oxidation layer
γ-Fe2O3 NPs
① Fenton-like reaction on surface
② Internal electron transfer
③ Excess Fe ions outward migration
④ Chemical composition change

Fig 10: Structural Modulation of Biogenic Iron Oxide Nanoparticles by Fungi and Implications for POD Activity

A. Enzyme-like Activities of Biogenic Ferrihydrite Nanoparticles (FN) by Fungi, (a, and b) Location of H_2O_2 (blue in a) and O_2^{*-} (brown in b) in hyphae shown by staining. (c and d) Progress and yield of the TMB colorimetric reaction catalyzed by 50 ng/mL HRP (c) in comparison to that catalyzed by FNs (e) Variation of peroxidase-like activity in culture with hematite, hematite + EPS, FNs, and the abiotic control (i.e., hematite exposed to a pH of 3.6; n = 3). (f) Relative abundance of H_2O_2 versus HO* in culture with and without FNs (n = 2 or 3)

B. Morphology of Fungal Hyphae in the Absence or Presence of Iron Nanoparticles (a and b) Cryo-SEM images of fungal biomass in the absence of hematite. Scale bar represents 5 µm. (c and d) Cryo-SEM images of fungal biomass in the presence of hematite (120-h cultivation). Scale bars represent 20 µm (C) or 5 µm . (e) Synchrotron radiation transmission X-ray microscopy (SR-TXM) image showing the presence of iron minerals, pores, and fungal hyphae in fungal-mineral aggregates (120 h). Scale bar represents 200 µm. (f) TEM image of fungal-mineral culture samples (120 h). Scale bar represents 5 µm.(g and h) Element-specific 3D volume renderings for the pre-absorption edge of (g) Fe at E1 = 706 eV and (h) the absorption edge of Fe at E2 = 710 eV.

Reprinted with permission from Ref[23]. Copyright 2020, Elsevier

C. Effect of time on the interaction of fungi and magnetite nanoparticles on the activity of the peroxidase. The upper left corner show the zero time and the downer right conner show the 120 h. The Corresponding X-ray photoelectron spectroscopy spectra of Fe 2p3/2 (b, d) and O 1s (c, e) at the cultivation times of (b, d) 0 h and (d, e) 120 h respectively.

Reprinted with permission from Ref[24], Copyright © 2021, John Wiley and Sons

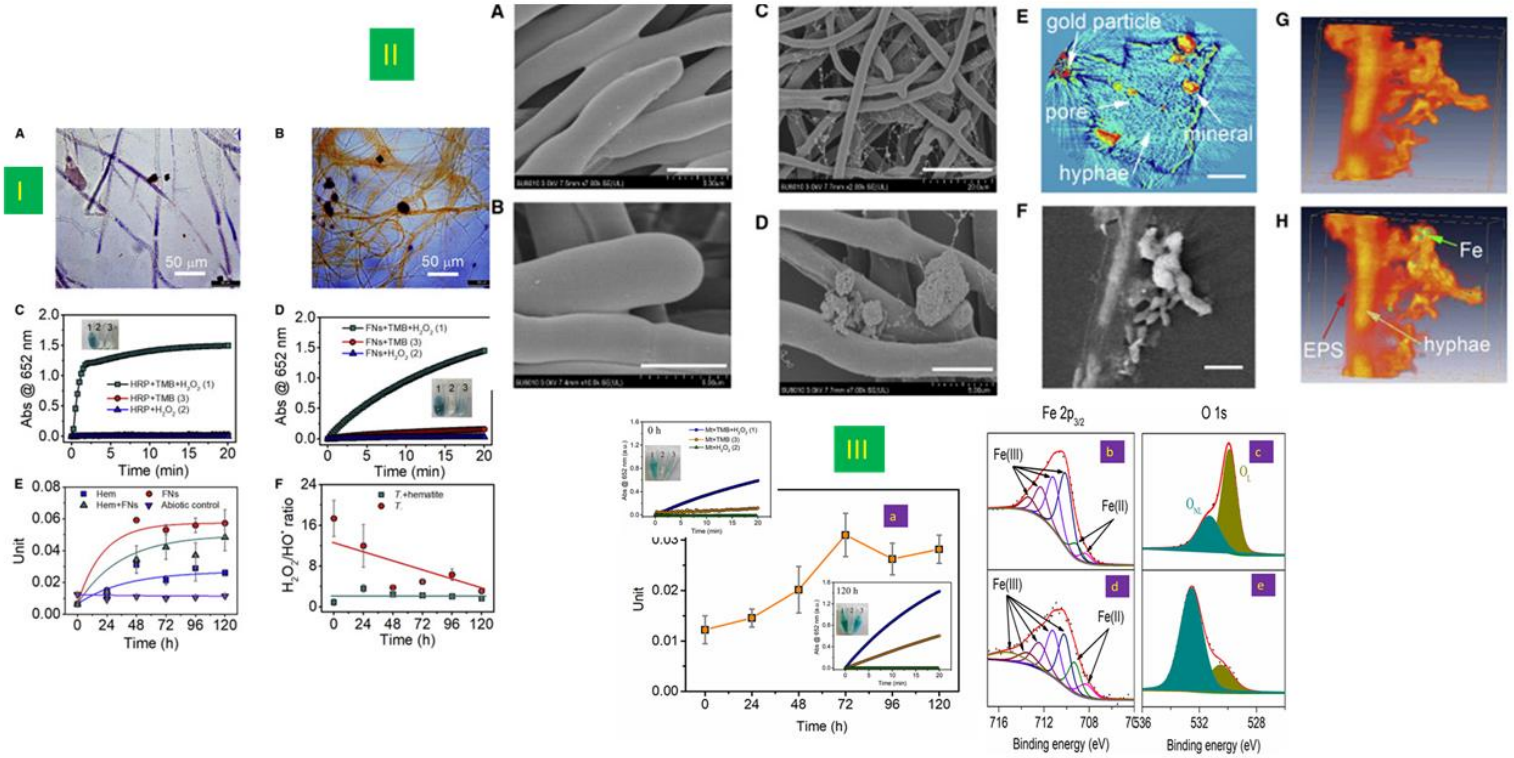

I
II
III
50 μm
Abs @ 652 nm
Time (min)
HRP+TMB+H_2O_2 (1)
HRP+TMB (3)
HRP+H_2O_2 (2)
FNs+TMB+H_2O_2 (1)
FNs+TMB (3)
FNs+H_2O_2 (2)
Unit
Time (h)
Hem
Hem+FNs
FNs
Abiotic control
H_2O_2/HO• ratio
T.+hematite
T.
gold particle
pore
mineral
hyphae
EPS
Fe
Fe $2p_{3/2}$
O 1s
Fe(III)
Fe(II)
O_{NL}
O_L
Binding energy (eV)

Fig 11. Magnetite Nanoparticles Structure and Electrical Characteristics

A. Surface plot of AC conductivity in function of temperature and frequency of of Fe_3O_4 [25]

B. Two conduction mechanism in different temperature and frequency regions[25],

Reprinted from Ref[25],

C. Response of Electronic resistivity of Fe_3O_4 under different pressure[26] Reprinted with permission from Ref[26], Copyright © 1997, John Wiley and Sons.

D. Dependence of the charge-ordering transition temperature for Fe_5O_6, Fe_4O_5, $MnFe_3O_5$, Fe_3O4, and $CaFe_3O_5$ on the minimal Fe−Fe distances in their octahedral iron chains[27]

Reprinted from Ref[27]

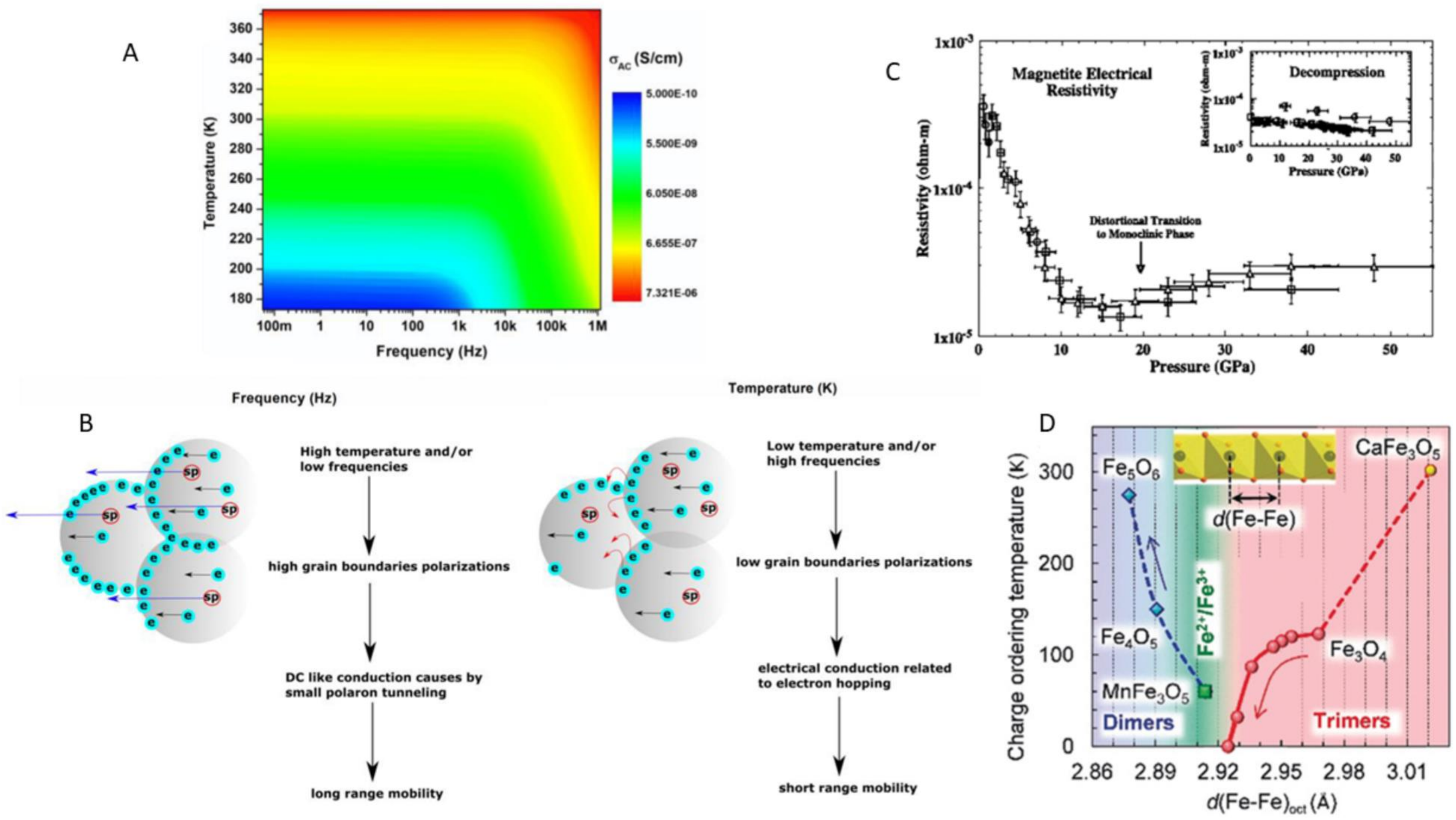
A
Temperature (K)
Frequency (Hz)
σAC (S/cm)
5.000E-10
5.500E-09
6.050E-08
6.655E-07
7.321E-06
C
Magnetite Electrical Resistivity
Decompression
Distortional Transition to Monoclinic Phase
Resistivity (ohm-m)
Pressure (GPa)
Frequency (Hz)
Temperature (K)
B
High temperature and/or low frequencies
high grain boundaries polarizations
DC like conduction causes by small polaron tunneling
long range mobility
Low temperature and/or high frequencies
low grain boundaries polarizations
electrical conduction related to electron hopping
short range mobility
D
Charge ordering temperature (K)
d(Fe-Fe)oct (Å)
d(Fe-Fe)
Fe5O6
Fe4O5
MnFe3O5
CaFe3O5
Fe3O4
Fe2+/Fe3+
Dimers
Trimers

Fig 12. Structure and Electronic Property of Three Iron Sulfides Nanoparticles

A. Crystal structure of Fe_3S_4 with the (001) and (111) planes outlined in blue and black, respectively. Sulfur atoms (yellow spheres) form a cubic close-packed lattice: 1/8 of the tetrahedral A sites are occupied by Fe^{3+} (blue spheres) and 1/2 of the octahedral B sites are occupied by Fe^{2+} and Fe^{3+} (red spheres) equally. The magnetic moments on the A and B sites are antiparallel and aligned along the [100] crystallographic axis (indicated by arrows).
B. Resistivity of greigite between 5 K and 300 K, and the corresponding contact geometry (inset), Reprinted with permission from Ref [28], Copyright © 2014, ACS
C. Crystal structures of Pyrite FeS_2 with the Fe-S octahedral and tetrahedral coordination. (a) The simple-cubic primitive unit cell of FeS_2. The transition metal atoms occupy the corners and the face centers of this cell. Each transition metal atom is in the center of a sulfur octahedron. (b) The transition metal octahedra are corner sharing in the pyrite structure. In addition, every sulfur is part of a dimer connecting neighboring octahedra., Reprinted with permission from permission from Ref[29], Copyright © 2020, APS
D. Calculated density of states from FeS_2. The inset shows a zoomed-in region near the Fermi level. Though the band gap may be lower, the onset of optical absorption is consistently measured to be 0.9 eV. Reprinted from Ref[30]
E. Structural phase diagram of FeS under different pressure and temperature conditions. The dashed phase boundaries (6.5–7.5 Gpa) demarcate a range where two phases coexist. Straight solid lines in the structures show the unit-cell as well as the octahedral coordination of Fe (small shaded circles) by S (larger open circles) atoms. Reprinted with permission from Ref[31], Copyright © 1999, APS

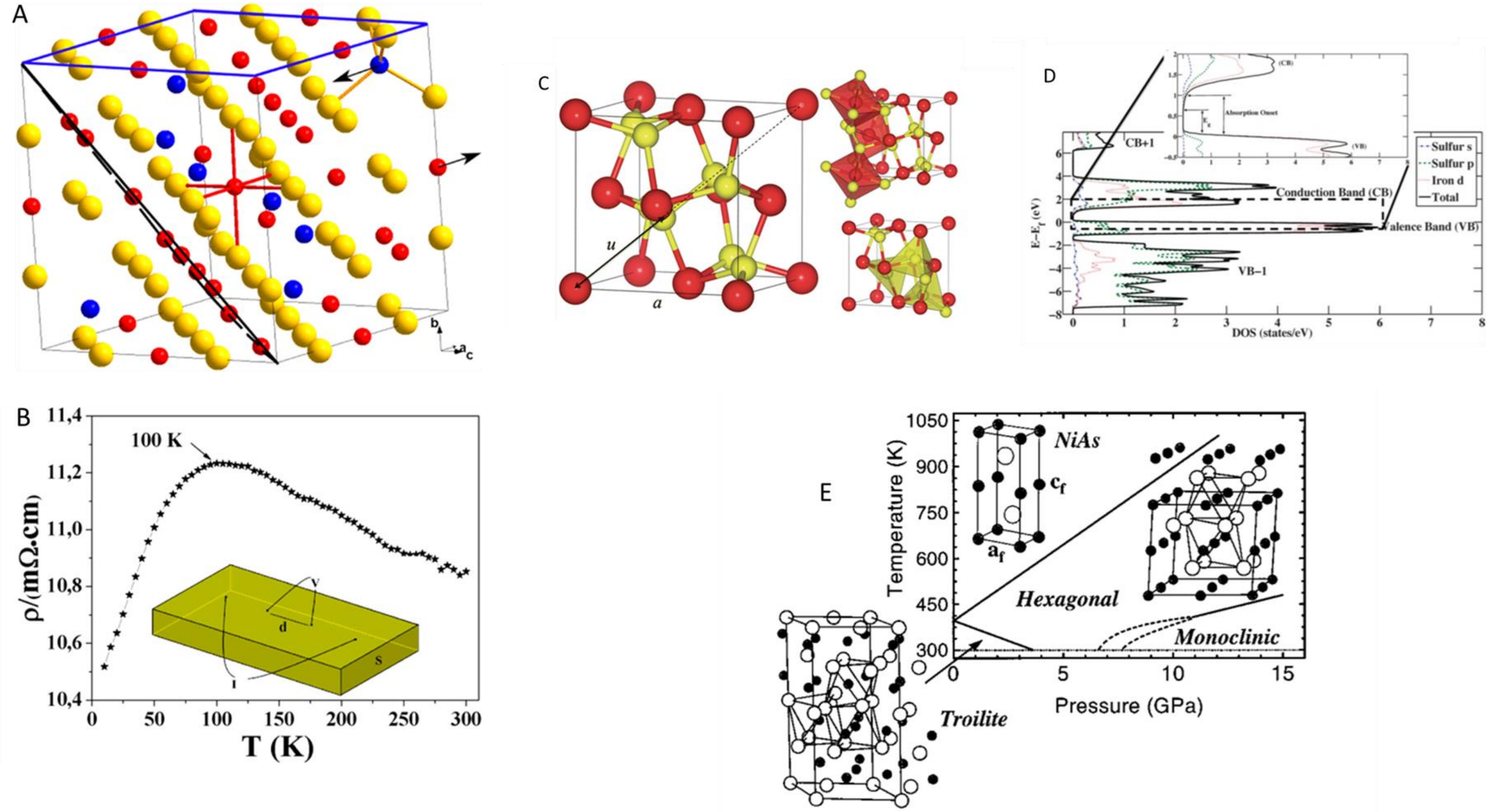
A
B
11,4
11,2
11,0
10,8
10,6
10,4
ρ/(mΩ•cm)
100 K
0
50
100
150
200
250
300
T (K)
C
u
a
D
E−E_f (eV)
DOS (states/eV)
CB+1
Conduction Band (CB)
Valence Band (VB)
VB−1
Sulfur s
Sulfur p
Iron d
Total
Absorption Onset
E
Temperature (K)
Pressure (GPa)
NiAs
Hexagonal
Monoclinic
Troilite

Fig. 13 Effect of Near-Infrared-II (NIR-II) Light on the Oxidoreductases Activity of MoO_3 Nanobelts

A. Digital photographs in powder and solution of inert MoO_3 and activated NH−MoO_3−x nanobelts
B. Electronic energy band of MoO_3 based on DFT model.
C. Electronic energy band of NH−MoO_3−x based on DFT model.
D. UV/Vis absorption spectra of ox TMB after different treatments (concentrations of NH−MoO_3−x: 50 μg mL^{-1}, H_2O_2: 1.0 mM, TMB: 1.0 mM, pH 6.5).
E. NIR-II laser-enhanced POD-like catalytic activity.
F. OXD-like catalytic activity testified by ESR spectra of NH−MoO_3−x in the presence of H_2O_2 using DMPO as the capture agent.
G. CAT-like catalytic activity of oxygen generation test results for NH−MoO_3−x@BSA in PBS with different pH values at 25 °C (concentration of NH−MoO_3−x: 50 μg mL⁻1, H_2O_2: 1 mM).
H. NIR-II laser effect on CAT-like catalytic activity.
I. Heating curves of NH−MoO_3−x@BSA with various concentrations by irradiation using a 1064 nm NIR-II laser for 10 min at 1.0 W cm^{-2}.

Reprinted from Ref [32]

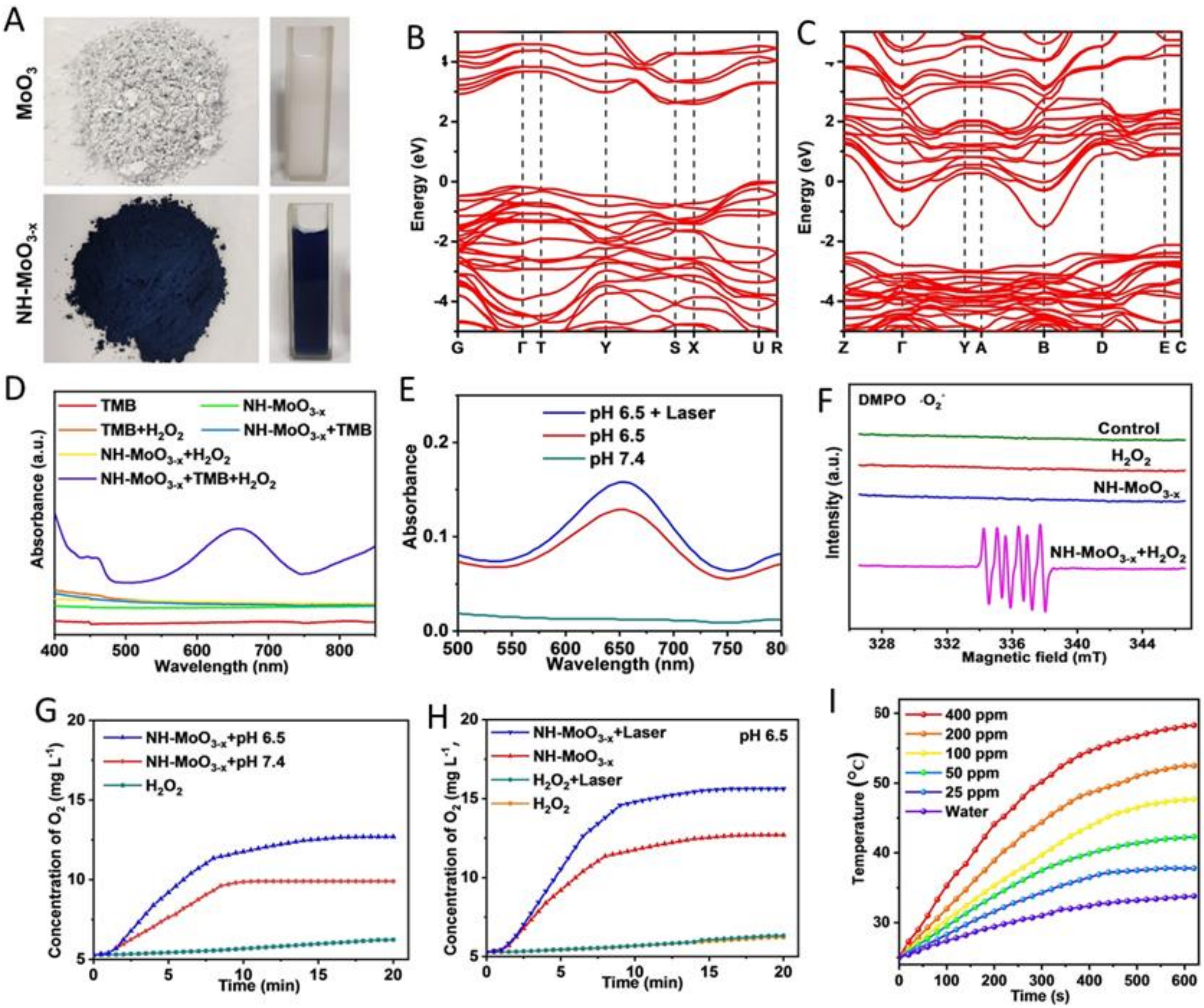

A
MoO3
NH-MoO3-x
B
Energy (eV)
G Γ T Y S X U R
C
Energy (eV)
Z Γ Y A B D E C
D
TMB
NH-MoO3-x
TMB+H2O2
NH-MoO3-x+TMB
NH-MoO3-x+H2O2
NH-MoO3-x+TMB+H2O2
Absorbance (a.u.)
Wavelength (nm)
E
pH 6.5 + Laser
pH 6.5
pH 7.4
Absorbance
Wavelength (nm)
F
DMPO ·O2-
Control
H2O2
NH-MoO3-x
NH-MoO3-x+H2O2
Intensity (a.u.)
Magnetic field (mT)
G
NH-MoO3-x+pH 6.5
NH-MoO3-x+pH 7.4
H2O2
Concentration of O2 (mg L-1)
Time (min)
H
NH-MoO3-x+Laser
pH 6.5
NH-MoO3-x
H2O2+Laser
H2O2
Concentration of O2 (mg L-1,
Time (min)
I
400 ppm
200 ppm
100 ppm
50 ppm
25 ppm
Water
Temperature (°C)
Time (s)

Fig. 14 Enzymatic Activities of Cyt-$CeVO_4$ Nanoparticles

A. (a). Superoxide production during CcO-like activity of $CeVO_4$ using WST-1. (b). Determination of H_2O_2 production using Amplex red during SOD-like reaction of $CeVO_4$ nanoparticles. (c). Measurement of hydroxyl radicals via fluorescence spectroscopy using TPA. (d). A comparison between CcO-like activity of $CeVO_4$ and other metal oxide nanoparticles. (e). Schematic representation of the 4-electron reduction of oxygen. (F). Current-potential curve obtained for Ch using rotating ring-disk voltammetry under O_2^- saturated conditions at different rotation rates. Inset: Koutecky-Levich plots at various rotation rates for Ch.
B. (a) The SOD activity of the inorganic $CeVO_4$ (b) Formation of H_2O_2 during SOD-like reaction of $CeVO_4$ nanoparticles. (c) Scheme depicting SOD-like activity. (d) A comparison of $CeVO_4$ nanozymes' activity with SOD-mimetic nanoparticles and other metal oxides.
C. Scheme $CeVO_4$ with Cyc c exhibiting Cyc c oxidase and SOD

Reprinted with permission from Ref. [33]. Copyright © 2019, John Wiley and Sons

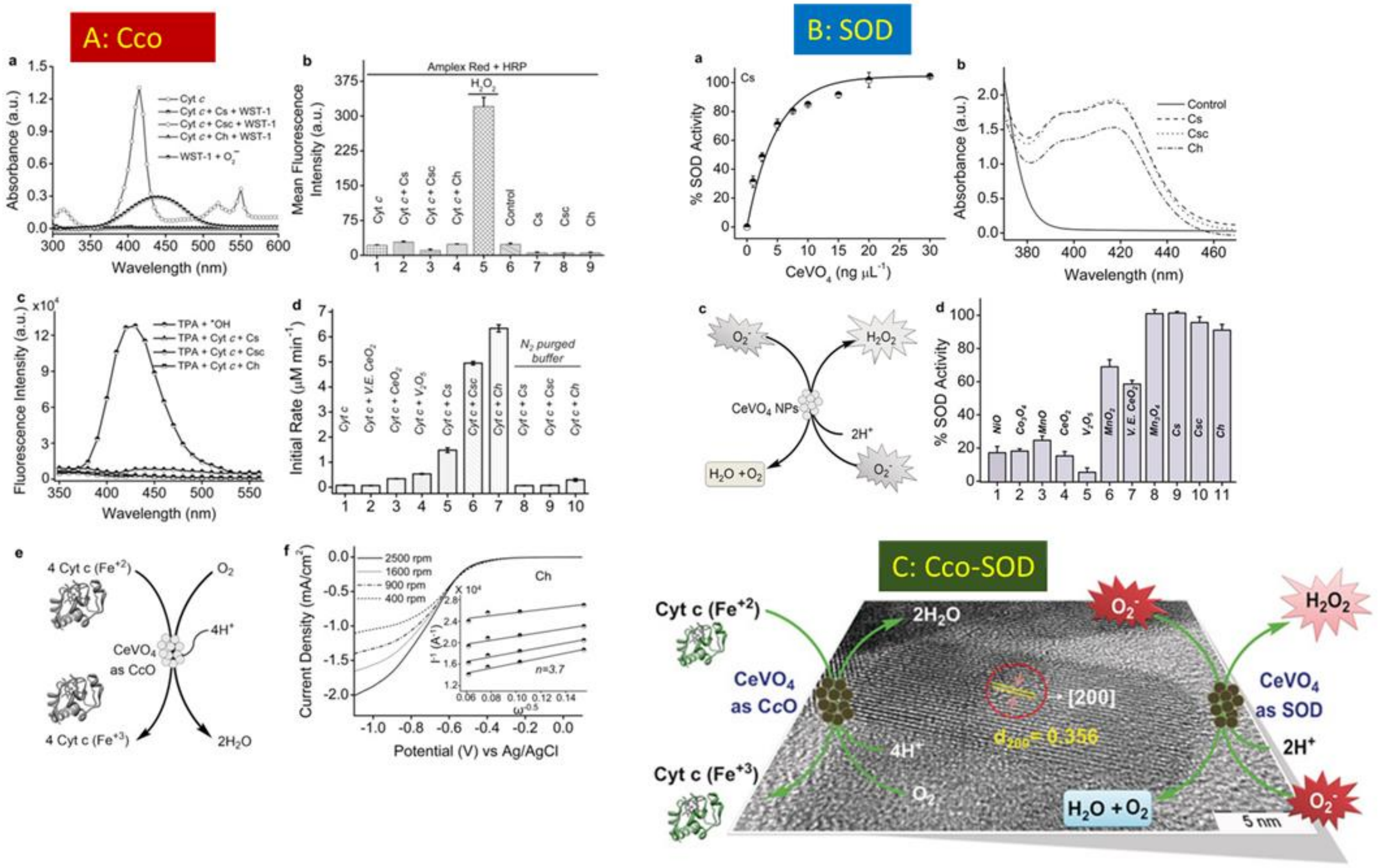
A: Cco
B: SOD
C: Cco-SOD
Absorbance (a.u.)
Wavelength (nm)
Cyt c
Cyt c + Cs + WST-1
Cyt c + Csc + WST-1
Cyt c + Ch + WST-1
WST-1 + O2−
Mean Fluorescence Intensity (a.u.)
Amplex Red + HRP
H2O2
Control
Fluorescence Intensity (a.u.)
TPA + •OH
TPA + Cyt c + Cs
TPA + Cyt c + Csc
TPA + Cyt c + Ch
Initial Rate (μM min−1)
N2 purged buffer
4 Cyt c (Fe+2)
4 Cyt c (Fe+3)
CeVO4 as CcO
4H+
O2
2H2O
Current Density (mA/cm2)
Potential (V) vs Ag/AgCl
2500 rpm
1600 rpm
900 rpm
400 rpm
n=3.7
% SOD Activity
CeVO4 (ng μL−1)
CeVO4 NPs
H2O + O2
H2O2
2H+
CeVO4 as SOD
d200 = 0.356
[200]
5 nm

Fig 15. Structures of Common and Non-conventional FeS Clusters.

A. [4Fe–4S] cluster, X in blue highlights a different cluster ligation, with terminal ($Fe–S^t$) and bridging ($Fe–S^b$) sulfides indicated in green and red, respectively;
B. [3Fe–4S] cluster;
C. [2Fe–2S] cluster including Rieske- (X = His, pink) and NEET-type (Y = His, red) clusters;
D. [4Fe–4S] cluster with bound S-adenosyl-L-methionine, O and NH_2 cluster coordination by SAM are marked in blue;
E. Siroheme [4Fe–4S] site of nitrite and sulfite reductases, the heme iron is displayed in dark yellow;
F. C cluster [Ni–4Fe–4S] in carbon monoxide dehydrogenase;
G. A cluster [2Ni–4Fe–4S] from acetyl-CoA synthase; the Ni ions in **F** and **G** are depicted in brown;
H. Nitrogenase FeMe-co [Me–7Fe–9S–C–(homocitrate)] M cluster, C (carbon) is represented in grey and Me (metal) in blue; nitrogenase NifB-co [8Fe–9S–C] L cluster, C (carbon) is marked in grey;
I. nitrogenase NifB-co [8Fe–9S–C] L cluster, C (carbon) is marked in grey;
J. [4Fe–4S]-S_n-[4Fe–4S] clusters (n = 1, 5) common to RimO, MiaB and double-cubane cluster proteins, S is depicted in blue;
M. [4Fe–3S] proximal cluster of the oxygen-tolerant membrane-bound [NiFe]-hydrogenase, the bound hydroxy (OH) ligand is depicted in blue;
N. H-cluster of [FeFe]-hydrogenases and its synthetic substitutions marked with X (black, Fe in the native form), Y (orange, S in the native form) and Z (grey, NH in the native form), CO and CN^- are depicted in red and blue respectively;
O. [4Fe–4S]–CN-[2Fe] cluster in HydF maturase, the bridging CN^- is shown in blue, CO and CN^- ligands are represented as in **N**;
P. [4Fe–4S]-(κ^3Cys)Fe(CN)$(CO)_2$ cluster in the [FeFe]-hydrogenase maturase HydG, a non-proteinaceous cysteine residue (κ^3-Cys) coordinates a 5th ion via its amino (NH_2, blue), carboxy (COO^-, blue) and thiolate (S^-, blue) functionalities, CO and CN^- ligands are depicted as in **N**;
Q. [4Fe–5S] cluster common to sulfurating enzymes, the extra SH is depicted in blue
R. Non-cubane [4Fe–4S] cluster of heterodisulfide reductase HdrB comprising a fused [2Fe–2S] and [3Fe–4S] cluster sharing one S and one Fe ion (depicted in blue).

Reprinted with permission from Ref.[34], Copyright © 2022, Elsevier

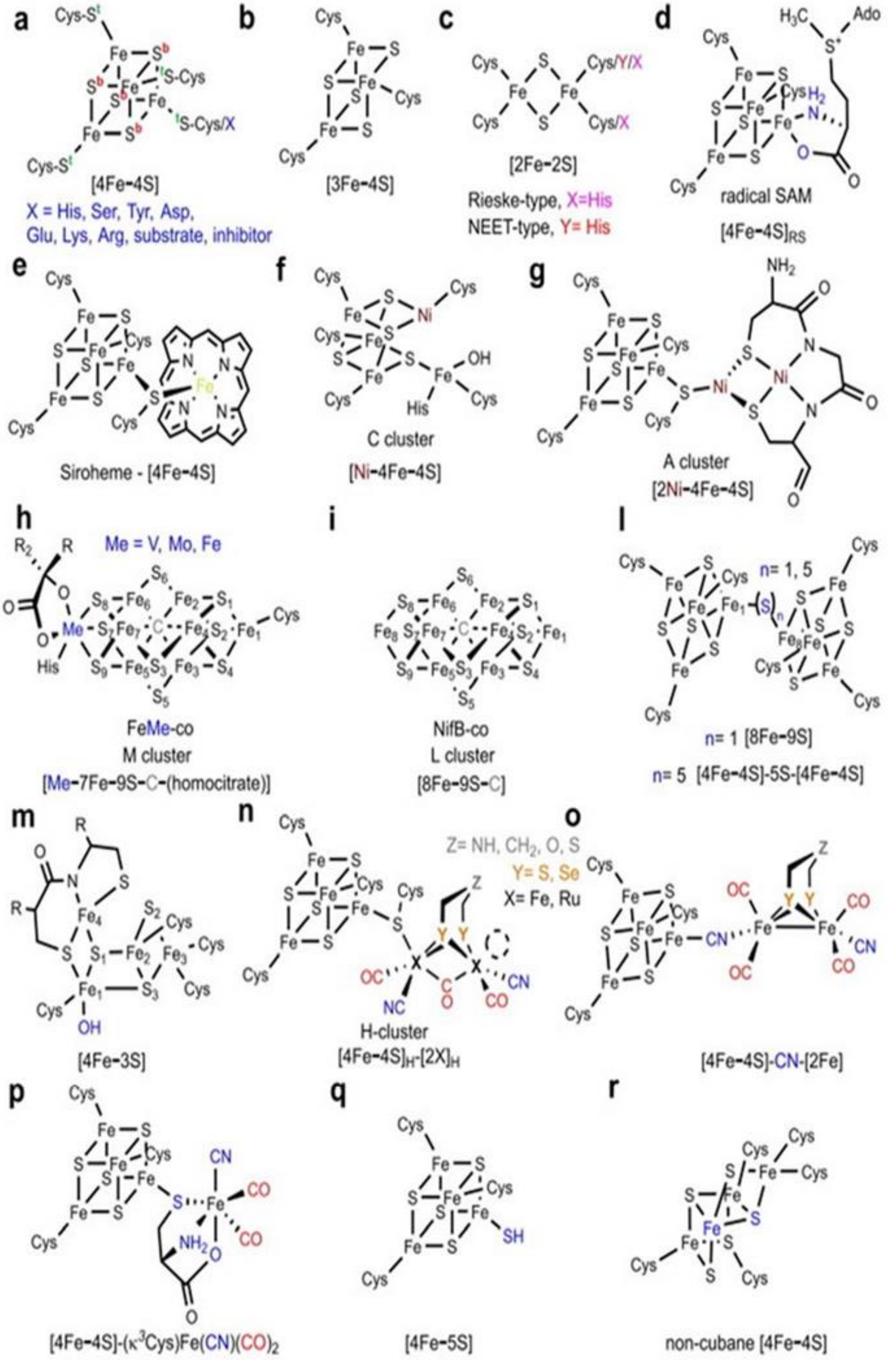

a
[4Fe-4S]
X = His, Ser, Tyr, Asp, Glu, Lys, Arg, substrate, inhibitor
b
[3Fe-4S]
c
[2Fe-2S]
Rieske-type, X=His
NEET-type, Y= His
d
radical SAM
[4Fe-4S]RS
e
Siroheme - [4Fe-4S]
f
C cluster
[Ni-4Fe-4S]
g
A cluster
[2Ni-4Fe-4S]
h
Me = V, Mo, Fe
FeMe-co
M cluster
[Me-7Fe-9S-C-(homocitrate)]
i
NifB-co
L cluster
[8Fe-9S-C]
l
n= 1, 5
n= 1 [8Fe-9S]
n= 5 [4Fe-4S]-5S-[4Fe-4S]
m
[4Fe-3S]
n
Z= NH, CH2, O, S
Y= S, Se
X= Fe, Ru
H-cluster
[4Fe-4S]H-[2X]H
o
[4Fe-4S]-CN-[2Fe]
p
[4Fe-4S]-(κ3Cys)Fe(CN)(CO)2
q
[4Fe-5S]
r
non-cubane [4Fe-4S]

Fig 16. Natural Iron Nanoparticles in the Earth Environments

A. Natural processes leading to the formation of nanoparticles in the environment, Reprinted with permission from Ref [35], Copyright © 2015, RSC
B. Magnetite nanoparticles in magnetosomes with elongated prismatic habits from magnetotactic A*lphaproteobacteria (a).* TEM image of a cell of a vibrioid MTB from Lake Mead, Nevada, containing a chain of elongated magnetosomes. (b). TEM image of two double chains of elongated magnetosomes from a freshwater coccus. c. High-resolution TEM image of a magnetosome from a freshwater coccus with (d) its selected-area electron diffraction pattern (in [1–10] orientation) and (e) a morphological model that consists of six large and six small dodecahedral faces, and smaller faces of the cube and octahedron. The elongation direction is [111]. Reprinted from Ref [36]
C. Iron oxide nanoparticles in Greenland and Antarctic Ice Sheets, Nanoparticulate ferrihydrite B5–10 nm in diameter has been identified. Images (b) and (c) are enlargements of (a), as indicated. The diffraction signal (d) shows some crystalline structure owing to possible impact of nano-clay particles, and potentially nano-hematite, but also the characteristic diffuse ferrihydrite rings are identifiable. EDS (e) analysis of the area further confirms Fe-dominated material. Reprinted with permission from Ref [37] Copyright © 2014, Spring
D. Iron oxide nanoparticles in street dust Urban Environment, (A) aggregate of spherical magnetite particles, (B) Fe-rich fly ash, (C) magnetite particle with an octahedral shape, (D) hematite, (E) ferrihydrite, (F) goethite. Reprinted with permission from Ref [38] Copyright © 2016, RSC
E. Iron nanoparticles from the hydrothermal vents, including various iron oxide and sulfides nanoparticles. They are important geochemical actors across naturally occurring marine redox gradients such as high-temperature hydrothermal vents and reducing sediments where metal turnover is orders of magnitude higher compared to other ocean zones. Reprinted from Ref [39]
F. Iron oxide nanoparticles are commonly presented in various soil. (Left) A cross section of loess deposit in Chinese Loess Plateau with loess layers (formed in dry and cold environment) and paleosol layers (formed in warm environment). warm environment. Paleosol layers display strong susceptibility signals than the loess layers due to presence of nano-maghemite. (Right). Transmission electron micrographs of magnetically-extracted, sub-micrometre pedogenic magnetite (likely partially oxidized towards maghemite), from palaeosol a) S5, b) and c) S1, Luochuan (Qinjiazhai section). Reprinted with permission from Ref[40], Copyright © 2016, Elsevier
G. Iron oxide nanoparticles with the various size, and sharp. Example morphologies of 1-dimensional and 3-dimensional NPs: **(A)** nanowires, **(B)** long nanotubes, (C) nanoneedles, **(D)** nanorods, **(E)** short nanotubes, **(F)** tube-in-tubes, and **(G)** nanorings. Reprinted from Ref [41]

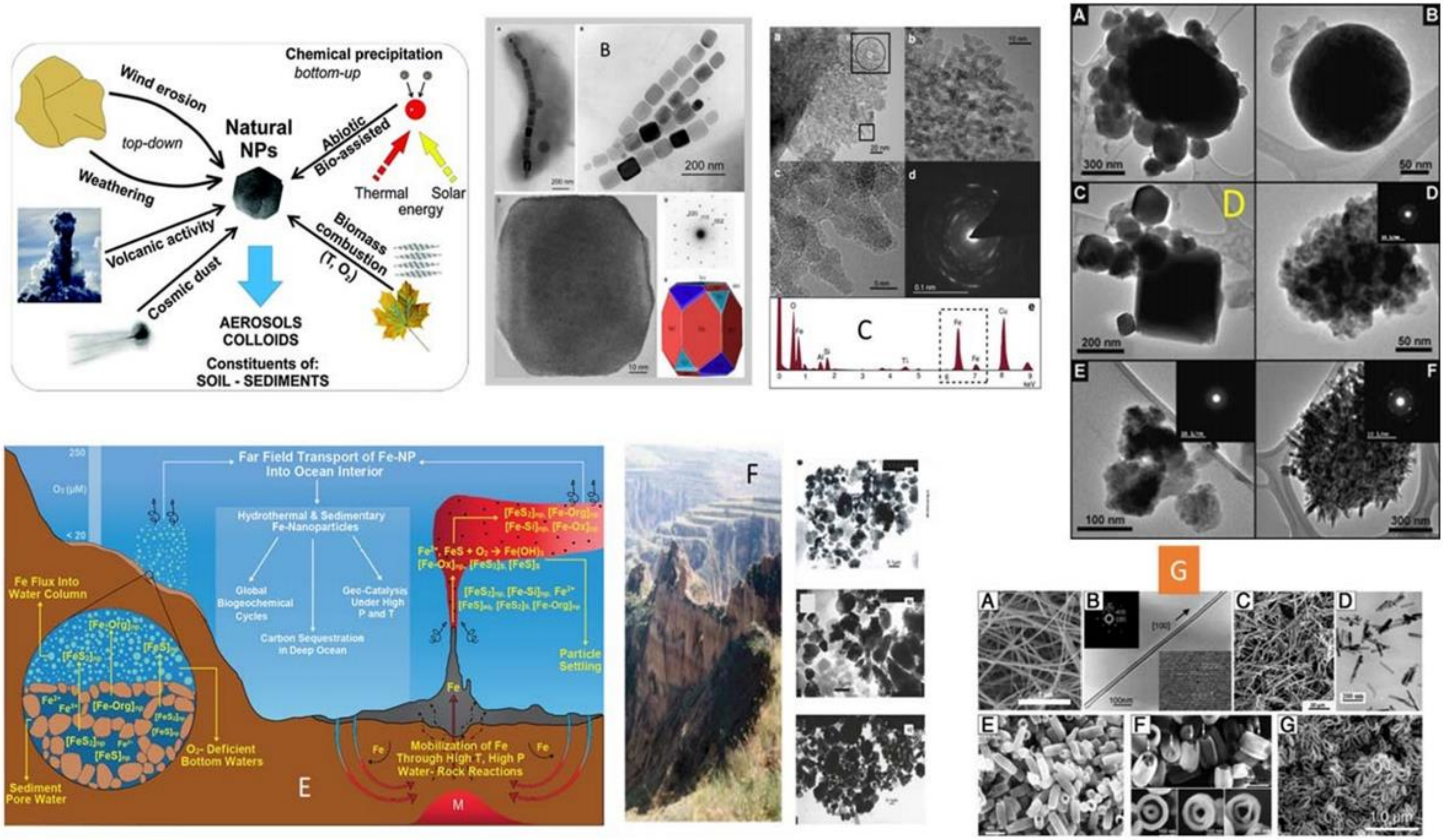
Wind erosion
top-down
Weathering
Volcanic activity
Cosmic dust
Natural NPs
Chemical precipitation
bottom-up
Abiotic
Bio-assisted
Thermal
Solar
energy
Biomass combustion
(T, O$_2$)
AEROSOLS
COLLOIDS
Constituents of:
SOIL - SEDIMENTS
B
200 nm
C
D
E
F
G
300 nm
200 nm
50 nm
100 nm
Far Field Transport of Fe-NP Into Ocean Interior
Hydrothermal & Sedimentary Fe-Nanoparticles
Global Biogeochemical Cycles
Carbon Sequestration in Deep Ocean
Geo-Catalysis Under High P and T
Fe Flux Into Water Column
Sediment Pore Water
O$_2$- Deficient Bottom Waters
Particle Settling
Mobilization of Fe Through High T, High P Water- Rock Reactions
M
10 μm

Reference

1 Huang, X. L. Hydrolysis of Phosphate Esters Catalyzed by Inorganic Iron Oxide Nanoparticles Acting as Biocatalysts. *Astrobiology* **18**, 294-310 (2018). https://doi.org:10.1089/ast.2016.1628

2 Huang, X.-L. What are inorganic nanozymes? Artificial or inorganic enzymes. *New J. Chem.* **46**, 15273-15291 (2022). https://doi.org:10.1039/D2NJ02088B

3 Huang, X. L. & Zhang, J. Z. Sediment-water exchange of dissolved organic phosphorus in Florida bay. 72-73 (University of Miami, Miami, FL, USA, 2007).

4 Huang, X. L. & Zhang, J. Z. Hydrolysis of glucose-6-phosphate in aged, acid-forced hydrolysed nanomolar inorganic iron solutions - An inorganic biocatalyst? *RSC Adv* **2**, 199-208 (2012). https://doi.org:10.1039/c1ra00353d

5 Zhang, R. *et al.* Unveiling the active sites on ferrihydrite with apparent catalase-like activity for potentiating radiotherapy. *Nano Today* **41**, 101317 (2021). https://doi.org:10.1016/j.nantod.2021.101317

6 Cornell, R. M. & Schwertmann, U. *The Iron Oxides: Structure, Properties, Reactions, Occurrence and Uses,* . Second edn, 664 (Wiley-VCH Verlag GmbH & Co. KGaA, 2003).

7 Tamura, H., Tanaka, A., Mita, K.-y. & Furuichi, R. Surface Hydroxyl Site Densities on Metal Oxides as a Measure for the Ion-Exchange Capacity. *J. Colloid Interface Sci.* **209**, 225-231 (1999). https://doi.org:10.1006/jcis.1998.5877

8 Dai, Z., Liu, S., Bao, J. & Ju, H. Nanostructured FeS as a mimic peroxidase for biocatalysis and biosensing. *Chemistry* **15**, 4321-4326 (2009). https://doi.org:10.1002/chem.200802158

9 Xu, Z. *et al.* Converting organosulfur compounds to inorganic polysulfides against resistant bacterial infections. *Nat. Commun.* **9**, 3713 (2018). https://doi.org:10.1038/s41467-018-06164-7

10 Shen, X., Wang, Z., Gao, X. & Zhao, Y. Density Functional Theory-Based Method to Predict the Activities of Nanomaterials as Peroxidase Mimics. *ACS Catal.* **10**, 12657-12665 (2020). https://doi.org:10.1021/acscatal.0c03426

11 Pan, Y. *et al.* Bacterial intracellular nanoparticles exhibiting antioxidant properties and the significance of their formation in ROS detoxification *Environ. Microbiol. Rep* **11**, 140-146 (2019). https://doi.org:10.1111/1758-2229.12733

12 Tang, Z., Wu, H., Zhang, Y., Li, Z. & Lin, Y. Enzyme-mimic activity of ferric nano-core residing in ferritin and its biosensing applications. *Anal. Chem.* **83**, 8611-8616 (2011). https://doi.org:10.1021/ac202049q

13 Ma, L. *et al.* A natural biogenic nanozyme for scavenging superoxide radicals. *Nat. Commun.* **15**, 233 (2024). https://doi.org:10.1038/s41467-023-44463-w

14 Karim, M. N. *et al.* Visible-Light-Triggered Reactive-Oxygen-Species-Mediated Antibacterial Activity of Peroxidase-Mimic CuO Nanorods. *ACS Appl. Nano Mat.* **1**, 1694-1704 (2018). https://doi.org:10.1021/acsanm.8b00153

15 Jolivet, J. P., Tronc, E. & Chanéac, C. Iron oxides: From molecular clusters to solid. A nice example of chemical versatility. *CR GEOSCI* **338**, 488-497 (2006). https://doi.org:10.1016/j.crte.2006.04.014

16 Michel, F. M. *et al.* The structure of ferrihydrite, a nanocrystalline material. *Science* **316**, 1726-1729 (2007). https://doi.org:10.1126/science.1142525

17 Manceau, A. & Combes, J. M. Structure of Mn and Fe oxides and oxyhydroxides: A topological approach by EXAFS. *Phys Chem Miner* **15**, 283-295 (1988). https://doi.org:10.1007/BF00307518

18 Li, Z. *et al.* Mechanism and kinetics of magnetite oxidation under hydrothermal conditions. *RSC Adv* **9**, 33633-33642 (2019). https://doi.org:10.1039/C9RA03234G

19 Usman, M. *et al.* Magnetite and Green Rust: Synthesis, Properties, and Environmental Applications of Mixed-Valent Iron Minerals. *Chem Rev* **118**, 3251-3304 (2018). https://doi.org:10.1021/acs.chemrev.7b00224

20 Usman, M. *et al.* FeII induced mineralogical transformations of ferric oxyhydroxides into magnetite of variable stoichiometry and morphology. *J Solid State Chem* **194**, 328-335 (2012). https://doi.org:10.1016/j.jssc.2012.05.022

21 Shu, Z. *et al.* Solar Irradiation Induced Transformation of Ferrihydrite in the Presence of Aqueous Fe2+. *Environ. Sci. Technol.* **53**, 8854-8861 (2019). https://doi.org:10.1021/acs.est.9b02750

22 Dong, H. *et al.* Depletable peroxidase-like activity of Fe3O4 nanozymes accompanied with separate migration of electrons and iron ions. *Nat. Commun.* **13**, 5365 (2022). https://doi.org:10.1038/s41467-022-33098-y

23 Yu, G. H. *et al.* Fungal Nanophase Particles Catalyze Iron Transformation for Oxidative Stress Removal and Iron Acquisition. *Curr. Biol.* **30**, 2943-2950.e2944 (2020). https://doi.org:10.1016/j.cub.2020.05.058

24 Chi, Z. L. *et al.* Intrinsic enzyme-like activity of magnetite particles is enhanced by cultivation with Trichoderma guizhouense. *Environ Microbiol* **23**, 893-907 (2021). https://doi.org:10.1111/1462-2920.15193

25 Radoń, A., Łukowiec, D., Kremzer, M., Mikuła, J. & Włodarczyk, P. Electrical Conduction Mechanism and Dielectric Properties of Spherical Shaped Fe_3O_4 Nanoparticles Synthesized by Co-Precipitation Method. *Materials* **11**, 735 (2018). https://doi.org:10.3390/ma11050735

26 Morris, E. R. & Williams, Q. Electrical resistivity of Fe3O4 to 48 GPa: Compression-induced changes in electron hopping at mantle pressures. *J. Geophys. Res. Solid Earth* **102**, 18139-18148 (1997). https://doi.org:10.1029/97JB00024

27 Ovsyannikov, S. V. *et al.* A Room-Temperature Verwey-type Transition in Iron Oxide, Fe5O6. *Angew. Chem. Int. Ed.* **59**, 5632-5636 (2020). https://doi.org:10.1002/anie.201914988

28 Li, G. *et al.* High-Purity Fe3S4 Greigite Microcrystals for Magnetic and Electrochemical Performance. *Chem. Mater.* **26**, 5821-5829 (2014). https://doi.org:10.1021/cm501493m

29 Day-Roberts, E., Birol, T. & Fernandes, R. M. Contrasting ferromagnetism in pyrite ${\mathrm{FeS}}_{2}$ induced by chemical doping versus electrostatic gating. *Physical Review Materials* **4**, 054405 (2020). https://doi.org:10.1103/PhysRevMaterials.4.054405

30 Sorenson, S. A., Patrow, J. G. & Dawlaty, J. M. Electronic Dynamics in Natural Iron Pyrite Studied by Broadband Transient Reflection Spectroscopy. *J. Phys. Chem. C* **120**, 7736-7747 (2016). https://doi.org:10.1021/acs.jpcc.5b11036

31 Takele, S. & Hearne, G. R. Electrical transport, magnetism, and spin-state configurations of high-pressure phases of FeS. *Phys. Rev. B* **60**, 4401-4403 (1999). https://doi.org:10.1103/PhysRevB.60.4401

32 Zhou, Z. *et al.* Intercalation-Activated Layered MoO3 Nanobelts as Biodegradable Nanozymes for Tumor-Specific Photo-Enhanced Catalytic Therapy. *Angew. Chem. Int. Ed.* **61** (2022). https://doi.org:10.1002/anie.202115939

33 Singh, N. & Mugesh, G. CeVO4 Nanozymes Catalyze the Reduction of Dioxygen to Water without Releasing Partially Reduced Oxygen Species. *Angew. Chem. Int. Ed.* **58**, 7797-7801 (2019). https://doi.org:10.1002/anie.201903427

34 Caserta, G. *et al.* Unusual structures and unknown roles of FeS clusters in metalloenzymes seen from a resonance Raman spectroscopic perspective. *Coord Chem Rev* **452**, 214287 (2022). https://doi.org:10.1016/j.ccr.2021.214287

35 Sharma, V. K., Filip, J., Zboril, R. & Varma, R. S. Natural inorganic nanoparticles--formation, fate, and toxicity in the environment. *Chem Soc Rev* **44**, 8410-8423 (2015). https://doi.org:10.1039/c5cs00236b

36 Pósfai, M., Lefèvre, C., Trubitsyn, D., Bazylinski, D. & Frankel, R. Phylogenetic significance of composition and crystal morphology of magnetosome minerals. *Front. Microbiol.* **4**, 344 (2013). https://doi.org:10.3389/fmicb.2013.00344

37 Hawkings, J. R. *et al.* Ice sheets as a significant source of highly reactive nanoparticulate iron to the oceans. *Nat. Commun.* **5**, 3929 (2014). https://doi.org:10.1038/ncomms4929

38 Yang, Y. *et al.* Nanoparticles in road dust from impervious urban surfaces: distribution, identification, and environmental implications. *Environ. Sci. Nano* **3**, 534-544 (2016). https://doi.org:10.1039/C6EN00056H

39 Yücel, M., Sevgen, S. & Le Bris, N. Soluble, Colloidal, and Particulate Iron Across the Hydrothermal Vent Mixing Zones in Broken Spur and Rainbow, Mid-Atlantic Ridge. *Front. Microbiol.* **12**, 631885 (2021). https://doi.org:10.3389/fmicb.2021.631885

40 Maher, B. A. Palaeoclimatic records of the loess/palaeosol sequences of the Chinese Loess Plateau. *Quat Sci Rev* **154**, 23-84 (2016). https://doi.org:https://doi.org/10.1016/j.quascirev.2016.08.004

41 Xie, W. *et al.* Shape-, size- and structure-controlled synthesis and biocompatibility of iron oxide nanoparticles for magnetic theranostics. *Theranostics* **8**, 3284-3307 (2018). https://doi.org:10.7150/thno.25220
